# Predictive Risk Factors at Admission and a “Burning Point” During Hospitalization Serve as Sequential Alerts for Critical Illness in Patients With COVID-19

**DOI:** 10.3389/fmed.2022.816314

**Published:** 2022-07-04

**Authors:** Zhengrong Yin, Mei Zhou, Juanjuan Xu, Kai Wang, Xingjie Hao, Xueyun Tan, Hui Li, Fen Wang, Chengguqiu Dai, Guanzhou Ma, Zhihui Wang, Limin Duan, Yang Jin

**Affiliations:** ^1^Key Laboratory of Pulmonary Diseases of National Health Commission, Department of Respiratory and Critical Care Medicine, Union Hospital, Tongji Medical College, Huazhong University of Science and Technology, Wuhan, China; ^2^State Key Laboratory of Environmental Health (Incubating), Department of Epidemiology and Biostatistics, School of Public Health, Tongji Medical College, Huazhong University of Science and Technology, Wuhan, China; ^3^Department of Scientific Research, Union Hospital, Tongji Medical College, Huazhong University of Science and Technology, Wuhan, China

**Keywords:** coronavirus disease 2019, critical illness onset, risk factor, “burning point”, predictive model, sequential alerts

## Abstract

**Background:**

We intended to establish a novel critical illness prediction system combining baseline risk factors with dynamic laboratory tests for patients with coronavirus disease 2019 (COVID-19).

**Methods:**

We evaluated patients with COVID-19 admitted to Wuhan West Union Hospital between 12 January and 25 February 2020. The data of patients were collected, and the illness severity was assessed.

**Results:**

Among 1,150 enrolled patients, 296 (25.7%) patients developed into critical illness. A baseline nomogram model consists of seven variables including age [odds ratio (OR), 1.028; 95% confidence interval (CI), 1.004–1.052], sequential organ failure assessment (SOFA) score (OR, 4.367; 95% CI, 3.230–5.903), neutrophil-to-lymphocyte ratio (NLR; OR, 1.094; 95% CI, 1.024–1.168), D-dimer (OR, 1.476; 95% CI, 1.107–1.968), lactate dehydrogenase (LDH; OR, 1.004; 95% CI, 1.001–1.006), international normalised ratio (INR; OR, 1.027; 95% CI, 0.999–1.055), and pneumonia area interpreted from computed tomography (CT) images (medium vs. small [OR, 4.358; 95% CI, 2.188–8.678], and large vs. small [OR, 9.567; 95% CI, 3.982–22.986]) were established to predict the risk for critical illness at admission. The differentiating power of this nomogram scoring system was perfect with an area under the curve (AUC) of 0.960 (95% CI, 0.941–0.972) in the training set and an AUC of 0.958 (95% CI, 0.936–0.980) in the testing set. In addition, a linear mixed model (LMM) based on dynamic change of seven variables consisting of SOFA score (value, 2; increase per day [I/d], +0.49), NLR (value, 10.61; I/d, +2.07), C-reactive protein (CRP; value, 46.9 mg/L; I/d, +4.95), glucose (value, 7.83 mmol/L; I/d, +0.2), D-dimer (value, 6.08 μg/L; I/d, +0.28), LDH (value, 461 U/L; I/d, +13.95), and blood urea nitrogen (BUN value, 6.51 mmol/L; I/d, +0.55) were established to assist in predicting occurrence time of critical illness onset during hospitalization.

**Conclusion:**

The two-checkpoint system could assist in accurately and dynamically predicting critical illness and timely adjusting the treatment regimen for patients with COVID-19.

## Introduction

The emergence of SARS-CoV-2 variants, with stronger transmissibility or ability to evade humoral immunity, has ushered in a new stage of the pandemic coronavirus disease 2019 (COVID-19) (1). Globally, 248,467,363 cumulative cases including 5,027,183 deaths have been confirmed until 5 November 2021, with hundreds of thousands of new cases increasing daily.^1^ In total, 5–20% of hospitalized patients with COVID-19 were admitted to the intensive care unit (ICU), with the mortality rate reportedly standing between 26 and 61.5% (2–6). The condition of patients with critical illness tends to deteriorate over a very short period of time, frequently leading to acute respiratory distress syndrome (ARDS) or multiple-organ failure, and even death (7, 8).

The ongoing pandemic with the high fatality rate of patients with critical illness necessitates the discovery of reliable prognostic predictors. So far, several studies (9–12) have reported predictive models for patients with critical illness and with COVID-19. Other studies suggested the prognostic value of longitudinal changes in clinical variables including ventilatory ratio (VR), platelet count, fibrinogen, and D-dimer (13, 14). However, these models had not integrated the baseline characteristics and the longitudinal analysis and were unable to predict disease progression during hospitalization. Here, we introduced a novel two-checkpoint prediction system based on both baseline characteristics at patient admission and longitudinal data collected during hospitalization. A crucial turning point—“burning point” was found before patients deteriorated to a critical condition (such as ICU admission), which was incorporated into this warning system. The two-checkpoint prediction system is a workable early warning system, including the first warning at admission and the second alert as early as 5 days before critical illness onset (CIO) to predict the occurrence and possible time of critical illness in patients with COVID-19.

## Materials and Methods

### Study Design and Participants

**FIGURE 1:**
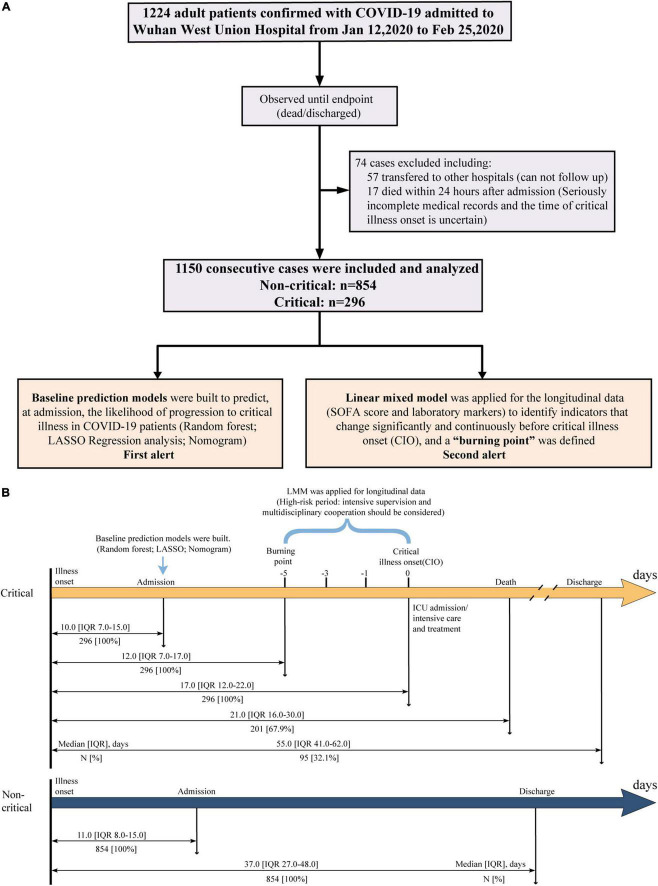
Study flowchart **(A)** and schematic diagram of timeline **(B)**.

A total of 1,224 Laboratory-confirmed adult patients with COVID-19 (≥ 18 years old) were consecutively admitted to Wuhan West Union Hospital between 12 January and 25 February 2020. Among which, 74 patients were excluded including 57 patients transferred to other hospitals and 17 patients who died within 24 h after admission. The remaining 1,150 participants were included in our study, and they all had a definite clinical outcome (death or discharge) as of early-May 2020 (the study flowchart is shown in Figure 1A). This study was approved by the Institutional Review Board of Medical Ethics Committee of Union Hospital, Huazhong University of Science and Technology (NO.0036). Written informed consent was waived by the Committee for this critical situation of emerging infectious diseases.

### Criteria and Definitions

The diagnosis and discharge criteria for COVID-19 were consistent with previous reports (10, 15). According to the interim criteria of WHO (16) and the guidelines by the National Health Commission (trial version 7.0), critical COVID-19 illness was evaluated retrospectively and confirmed based on respiratory infection, including one of the following: (1) ARDS needing mechanical ventilation, (2) sepsis leading to life-threatening organ dysfunction, and (3) septic shock. Otherwise, the patients were identified as non-critical patients. The CIO was recorded as the beginning time of moderate/severe ARDS requiring mechanical ventilation, or the time point at which sepsis caused the life-threatening multiple organ dysfunction or the septic shock developed or the patient was admitted to ICU. We introduced a new concept—“burning point” and defined it as a critical turning point at which the condition was exacerbated before CIO, and some indicators started to change significantly and continuously. The period from the burning point to CIO was deemed a high-risk period for CIO. The first alert comes from the baseline warning system at admission, and the second alert comes from the “burning point” warning system during hospitalization. ARDS was diagnosed according to the Berlin definition (17). Sepsis and septic shock were defined based on the 2016 Third International Consensus Definition (18). The sequential organ failure assessment (SOFA) score was calculated as previously reported (19). Definitions of various organ injuries were described in Supplementary Materials.

### Data Collection

A total of 87 baseline variables, covering demographics, comorbidities, symptoms, laboratory findings, imaging features, SOFA score, and admission time, were collected from electronic medical documents. The baseline computed tomography (CT) images were interpreted independently by two senior radiologists experienced in chest radiology. For all participants, the SOFA score and all laboratory data (47 items in total) were recorded from admission to discharge or death. At least two experienced doctors carefully went through the medical records of each critical patient to determine the time of the CIO. All of these data were summed up in a standardized form.

### Descriptive Analysis

Categorical variables were presented as frequencies (*n*) and percentages (%). The continuous variables with normal or non-normal distribution were expressed as mean ± standard deviation (*SD*) or median [interquartile range (IQR)]. To compare the differences in baseline variables between critical and non-critical participants, we used the independent sample *t*-test or Mann–Whitney *U*-test for continuous variables, χ^2^-test, Fisher’s exact test, or Mann–Whitney *U*-test were employed for categorical (binary or ordinal) variables wherever appropriate.

### Variable Selection and Model Construction

To ensure the data integrity and avoid potential selection bias, variables or patients with a missing rate of less than 40% were all included. As a result, 81 variables and 1,118 patients remained. The random forest machine learning method was employed to impute the missing values (20). Principal component analysis (PCA) was then conducted by using the R package “factoextra” (21) to evaluate the distribution of patients and the most relevant variables for critical illness. No cases were labeled as outliers and excluded in this process. Thereafter, a total of 1,118 remaining patients were randomized into training and testing sets at a ratio of 7:3 (Training set, *N* = 783 [Non-critical/Critical: 587/196]; Testing set, *N* = 335 [Non-critical/Critical: 241/94]).

Three predictive models [i.e., the machine-learning-based random forest, the least absolute shrinkage and selection operator (LASSO) logistic regression, and the multivariable logistic regression models] were built to predict, at admission, the likelihood of progression to critical illness in patients with COVID-19. Briefly, we chose the predictors selected by both the random forest and LASSO regression models as candidate risk factors to conduct a multivariable logistic regression analysis and then developed a nomogram scoring system. Finally, the three models were further compared and validated (Supplementary Tables S1–6 and Supplementary Figures S1–4).

### Longitudinal Data Analysis

The SOFA score and 46 laboratory markers (47 indicators in total) were recorded successively in all the 1,150 hospitalized patients with COVID-19. To find out the indicators that changed significantly during the period of critical illness development, the linear mixed model (LMM) implemented in the R package “lme4” (22) was used to explore the association between time and indicators by taking age and sex as fixed effects.

All tests were two-sided, and a *p-*value less than 0.05 was considered statistically significant. R software (version 3.6.2, R Foundation) was used for all analyses.

## Results

### Features and Outcomes of Patients With Non-critical and Critical COVID-19

In our study, we collected data from 1,150 consecutively admitted patients. All the participants were studied until discharge or death (Figure 1A). Among them, 296 of 1,150 patients (25.7%) were identified to be critically ill. As shown in Table 1, the overall mortality was 17.5% (201/1,150), while up to 67.9% in critically ill. All non-critical patients were discharged, and their hospital stay time was significantly shorter than critical patients discharged (23.0 vs. 43.0, *p* < 0.0001). The median age of non-critical and critical patients was 59.0 (IQR, 48.0–68.0) and 68.0 (61.0–76.0) years, respectively. There were more male patients in the critical group than in the non-critical group (64.2% vs. 46.6%, *p* < 0.0001). Over half of the patients had fever (81.4%) and cough (68.3%) at admission. In total, 778 (68.7%) patients had at least one comorbidity, including hypertension (33.6%), diabetes (20.4%), and coronary heart disease (10.9%) as the top three comorbidities. Sepsis (48.1%) was the most frequent complication, followed by acute liver injury (31.4%), ARDS (31.1%), acute cardiac injury (13.5%), and acute kidney injury (13.1%). The frequencies of complications were significantly higher in critical patients (all *p* < 0.0001). Both the SOFA score at admission (3.00 vs. 1.00, *p* < 0.0001) and the highest SOFA score during hospitalization (6.00 vs. 1.00, *p* < 0.0001) were significantly higher in critical patients. The baseline CT features and laboratory findings among critical and non-critical patients were also summarized in Table 1. The time from illness onset to admission, “burning point,” CIO, death, or discharge was listed in Figure 1B.

**TABLE 1:** Baseline characteristics and outcomes of critical and non-critical patients with COVID-19.

Variables	All patients, (*n* = 1,150)	Non-critical patients, *(n* = 854)	Critical patients, (*n* = 296)	*p-*value
**Demographics**				
Age, median (IQR), years	62.0 (52.0, 70.0)	59.0 (48.0, 68.0)	68.0 (61.0, 76.0)	<0.0001
Sex				
Male, *n* (%)	588 (51.1)	398 (46.6)	190 (64.2)	<0.0001
Female, *n* (%)	562 (48.9)	456 (53.4)	106 (35.8)	
**Initial symptoms, *n*/N (%)**				
Fever	912/1,120 (81.4)	688/844 (81.5)	224/276 (81.2)	0.895
Highest temperature, median (IQR),°C	38.20 (37.50, 39.00)	38.00 (37.50, 39.00)	38.50 (37.63, 39.00)	0.065
Sore throat	44/1,091 (4.0)	34/828 (4.1)	10/263 (3.8)	0.817
Fatigue	531/1,104 (48.1)	390/835 (46.7)	141/269 (52.4)	0.150
Myalgia	238/1,096 (21.7)	192/832 (23.1)	46/264 (17.4)	0.057
Cough	759/1,113 (68.3)	576/842 (68.4)	184/271 (67.9)	0.868
Sputum production	361/1,104 (32.7)	262/836 (31.3)	99/268 (36.9)	0.095
Chest tightness	348/1,104 (31.5)	241/835 (28.9)	107/269 (39.8)	0.0008
Dyspnea	307/1,099 (27.9)	184/831 (22.1)	123/268 (45.9)	<0.0001
Running nose	22/1,095 (2.0)	14/828 (1.7)	8/267 (3.0)	0.191
Vomiting	83/1,100 (7.5)	71/833 (8.5)	12/267 (4.5)	0.036
Nausea	71/1,100 (6.5)	60/838 (7.2)	11/262 (4.2)	0.083
Diarrhea	171/1,103 (15.5)	131/834 (15.7)	40/269 (14.9)	0.800
Headache	69/1,098 (6.3)	59/828 (7.1)	10/270 (3.7)	0.052
Asymptomatic	13/1,120 (1.2)	12/870 (1.4)	1/250 (0.4)	0.291
**Comorbidities, *n*/N (%)**				
Hypertension	381/1,133 (33.6)	249/837 (29.7)	132/296 (44.6)	<0.0001
Diabetes	231/1,133 (20.4)	139/837 (16.6)	92/296 (31.1)	<0.0001
Coronary heart disease	123/1,133 (10.9)	76/837 (9.1)	47/296 (15.9)	0.0012
Cerebrovascular disease	49/1,133 (4.3)	16/837 (1.9)	33/296 (11.1)	<0.0001
Malignancy	64/1,133 (5.6)	40/837 (4.8)	24/296 (8.1)	0.033
Chronic bronchitis	27/1,133 (2.4)	21/837 (2.5)	6/296 (2.0)	0.640
Asthma	14/1133 (1.2)	12/837 (1.4)	2/296 (0.7)	0.479
Chronic obstructive pulmonary disease	19/1,133 (1.7)	9/837 (1.1)	10/296 (3.4)	0.017
Kidney disease	50/1,133 (4.4)	32/837 (3.8)	18/296 (6.1)	0.104
Liver disease	54/1,133 (4.8)	45/837 (5.4)	9/296 (3.0)	0.105
Others	360/1,133 (31.8)	258/837 (30.8)	102/296 (34.5)	0.248
**Number of comorbidities, *n*/N (%)**				
≥1	778/1,133 (68.7)	524/837 (62.6)	254/296 (85.8)	<0.0001
≥2	392/1,133 (34.6)	250/837 (29.9)	142/296 (48.0)	
≥3	150/1,133 (13.2)	94/837 (11.2)	56/296 (18.9)	
≥4	36/1,133 (3.2)	18/837 (2.2)	18/296 (6.1)	
**Complications, *n*/N (%)**				
Sepsis	553/1,149 (48.1)	258/854 (30.2)	295/295 (100)	<0.0001
Acute respiratory distress syndrome	358/1,150 (31.1)	66/854 (7.7)	292/296 (98.6)	<0.0001
Acute liver injury	361/1,149 (31.4)	187/854 (21.9)	174/295 (59.0)	<0.0001
Acute cardiac injury	155/1,149 (13.5)	31/854 (3.6)	124/295 (42.0)	<0.0001
Acute kidney injury	150/1,149 (13.1)	42/854 (4.9)	108/295 (36.6)	<0.0001
**Baseline CT findings, *n*/N (%)**				
**Pneumonia area (Lesion ratio to lung)**				
Small area (≤ 35%)	485/1,109 (43.7)	450/849 (53.0)	35/260 (13.5)	<0.0001
Medium area (35–65%)	493/1,109 (44.5)	347/849 (40.9)	146/260 (56.2)	
Large area (> 65%)	131/1,109 (11.8)	52/849 (6.1)	79/260 (30.4)	
**Uni-/bilateral pneumonia**				
Unilateral pneumonia	164/1,109 (14.8)	137/849 (16.1)	27/260 (10.4)	0.022
Bilateral pneumonia	945/1,109 (85.2)	712/849 (83.9)	233/260 (89.6)	
**Features and location of pulmonary lesions**				
Central	2/1,109 (0.2)	1/849 (0.1)	1/260 (0.4)	<0.0001
Peripheral	282/1,109 (25.4)	240/849 (28.3)	42/260 (16.2)	
Both	825/1,109 (74.4)	608/849 (71.6)	217/260 (83.5)	
Consolidation	858/1,109 (77.4)	623/849 (73.4)	235/260 (90.4)	<0.0001
Patchy exudation	1,030/1,109 (92.9)	784/849 (92.3)	246/260 (94.6)	0.218
Ground-glass opacity	964/1,109 (86.9)	722/849 (85.0)	242/260 (93.1)	0.0008
White lung	42/1,109 (3.8)	9/849 (1.1)	33/260 (12.7)	<0.0001
Pleural effusion	152/1,109 (13.7)	98/849 (11.5)	54/260 (20.8)	0.0002
Lymph node enlargement	91/1,109 (8.2)	73/849 (8.6)	18/260 (6.9)	0.389
**SOFA score, median (IQR)**				
SOFA score at admission	1.00 (0.00, 2.00)	1.00 (0.00, 1.00)	3.00 (2.00, 4.00)	<0.0001
Highest SOFA score during hospitalization	1.00 (1.00, 3.00)	1.00 (0.00, 2.00)	6.00 (4.00, 11.00)	<0.0001
**Representative baseline laboratory findings, median (IQR) or mean ( ± *SD*)**				
Neutrophil-to-lymphocyte ratio	3.80 (2.23, 6.93)	3.18 (1.99, 5.24)	8.48 (5.02, 13.07)	<0.0001
Lactate dehydrogenase, U/L	256.00 (195.00, 362.75)	234.00 (187.00, 308.00)	412.00 (301.00, 558.50)	<0.0001
D-dimer, μg/mL	0.51 (0.25, 1.00)	0.44 (0.22, 0.87)	0.83 (0.42, 1.73)	<0.0001
International normalized ratio	1.02 (0.95, 1.10)	1.01 (0.95, 1.07)	1.08 (1.00, 1.19)	<0.0001
**Outcomes and timeline**				
Discharged, *n*/N (%)	949/1,150 (82.5)	854/854 (100.0)	95/296 (32.1)	<0.0001
Deceased, *n*/N (%)	201/1,150 (17.5)	0/854 (0.0)	201/296 (67.9)	
Time from illness onset to admission, median (IQR), days	11.0 (7.0, 15.0)	11.0 (8.0, 15.0)	10.0 (7.0, 15.0)	0.045
Time from admission to death, median (IQR), days	10.0 (6.0, 18.0)	–	10.0 (6.0, 18.0)	–
Time from admission to discharge, median (IQR), days	25.0 (17.0, 37.0)	23.0 (16.0, 34.0)	43.0 (31.0, 50.0)	<0.0001

*Data were presented as n/N (%), median (IQR) or mean ( ± SD). p-values were calculated by Mann–Whitney U-test, χ^2^-test, or Fisher’s exact, if not specified.*

*IQR, interquartile range; SD, standard deviation; SOFA, sequential organ failure assessment.*

### Baseline Predictor Selection in the Training Set

**FIGURE 2:**
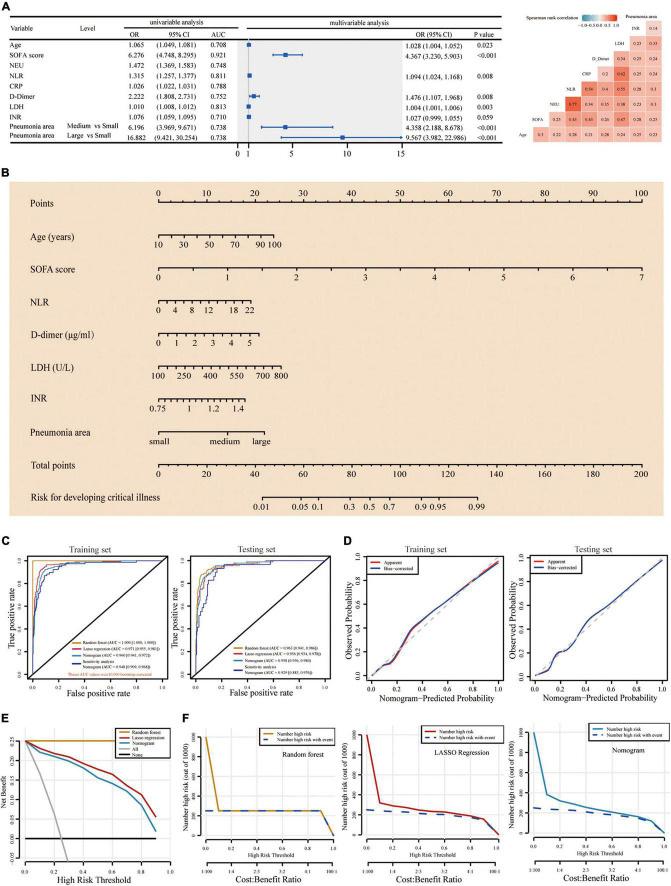
Construction of and comparison among the three baseline predictive models. **(A)** Univariable logistic analysis of the nine variables selected by both random forest and LASSO predictive models. Multivariable logistic analysis of the seven remained variables, with NEU and CRP excluded according to the spearman rank correlation for the nine variables. **(B)** The predictive nomogram scoring system was developed in the training set, with age, SOFA score, NLR, D-dimer, LDH, INR, and pneumonia area interpreted from CT images incorporated. **(C)** Four ROC plots for three predictive models (random forest, LASSO regression analysis-based model, and multivariable logistic regression analysis-based nomogram) and sensitivity analysis of nomogram, in training and testing set, respectively. The AUCs and 95% CIs for these models were computed with 10,000 bootstrap resample in the training set. **(D)** Calibration plots of the nomogram in training and testing set. The ideal calibration curve (gray dotted line), raw calibration curve (red curve), and the bootstrap-corrected calibration curve (blue curve) were displayed. **(E)** DCA comparing the clinical utility of the random forest (yellow line), LASSO (red line), and nomogram (ocean blue line) models. The gray line and horizontal solid black line reflect the corresponding net benefit if some intervention strategies conducted in all or no patients across the full range of threshold probabilities at which a patient would undergo special intervention to avoid critical illness. **(F)** Clinical impact curves of random forest (yellow line), LASSO regression (red line), and nomogram (ocean blue line)-based predictive model. They were evaluated by the predictive performance of risk stratification for 1,000 people and the corresponding cost–benefit ratio. The yellow, red, and ocean blue lines represent the number of people classified as high risk by each model under different threshold probability; the blue dotted curve is the number of truly positive people under different threshold probability. LASSO, least absolute shrinkage and selection operator; LDH, lactate dehydrogenase; SOFA, sequential organ failure assessment; ROC, receiver operating characteristic curve; NLR, neutrophil-to-lymphocyte ratio; DCA, Decision curve analysis; AUC, area under the curve; CRP, C-reactive protein; NEU, neutrophil.

The baseline laboratory results of critical and non-critical patients with COVID-19 were shown in Table 1 and Supplementary Table S7. The random forest and LASSO regression analysis were conducted in the training set, respectively, with the top 20 important variables remaining after random forest analysis and 19 variables selected by the latter (Supplementary Tables S3, 4 and Supplementary Figure S2). The nine variables selected by both random forest and LASSO regression models were used in the subsequent multivariable logistic regression analysis, with two variables [neutrophils (NEUs) and C-reactive protein (CRP)] excluded for their high correlation, respectively, with neutrophil-to-lymphocyte ratio (NLR) and lactate dehydrogenase (LDH) and relatively lower area under the curve (AUC) value (Figure 2A). These seven variables included age (odds ratio [OR], 1.028; 95% confidence interval [CI], 1.004–1.052; *p* = 0.023), SOFA score (OR, 4.367; 95% CI, 3.230–5.903; *p* < 0.001), NLR (OR, 1.094; 95% CI, 1.024–1.168; *p* = 0.008), D-dimer (OR, 1.476; 95% CI, 1.107–1.968; *p* = 0.008), LDH (OR, 1.004; 95% CI, 1.001–1.006; *p* = 0.003), INR (OR, 1.027; 95% CI, 0.999–1.055; *p* = 0.059), and pneumonia area interpreted from CT images (medium vs. small [OR, 4.358; 95% CI, 2.188–8.678; *p* < 0.001]; and large vs. small [OR, 9.567; 95% CI, 3.982–22.986; *p* < 0.001]) (Figure 2A).

### First Alert: A Baseline Nomogram Model for the Prediction at the Admission of the Risk for Critical Illness

For easy clinical application, we developed a nomogram scoring system in the training set based on the seven aforementioned variables to predict, at admission, the likelihood of progression to critical illness in patients with COVID-19, which could figure out the predicted probability of a patient developing critical illness during hospitalization (Figure 2B). Internal 10,000 bootstrap resamples exhibited that the nomogram had good distinguishing power, with its AUC reaching 0.960 (95%CI, 0.941–0.972), comparable to the other two models (random forest: 1.000 [95%CI, 1.000–1.000] and LASSO regression: 0.971 [95%CI, 0.955–0.981]) (Figure 2C). The non-parametric bootstrap test in the validation dataset showed that there were no statistically significant differences in AUCs among the three models (all *p* > 0.05) (Supplementary Table S5). In addition, the calibration curve of the nomogram model suggested that the predictive probability for critical illness fitted very well with the actual probability in both the training and the testing set (Figure 2D). In the testing set, the H–L test further confirmed the good performance of this model (*p* = 0.863) (Supplementary Table S6 and Supplementary Figure S3). Importantly, we performed a sensitivity analysis for this nomogram model based on the variables without missing values, yielding an AUC of 0.948 (*p* = 0.43) and 0.929 (*p* = 0.26), respectively, in the training and testing set (Figure 2C and Supplementary Table S5). As shown in Figures 2E,F, the decision curve analysis (DCA) and clinical impact curves proved that this nomogram worked well in supporting clinical decision-making, not much different from the other two predictive models. In addition, the nomogram scoring system was finally transformed into an online predictive tool: https://hust-covid19.shinyapps.io/Critical-illness-Predictive-Tool/ (Supplementary Figure S4).

### Differences in Dynamic Changes of Sequential Organ Failure Assessment Score and Laboratory Markers Between Critical and Non-critical Patients

**FIGURE 3:**
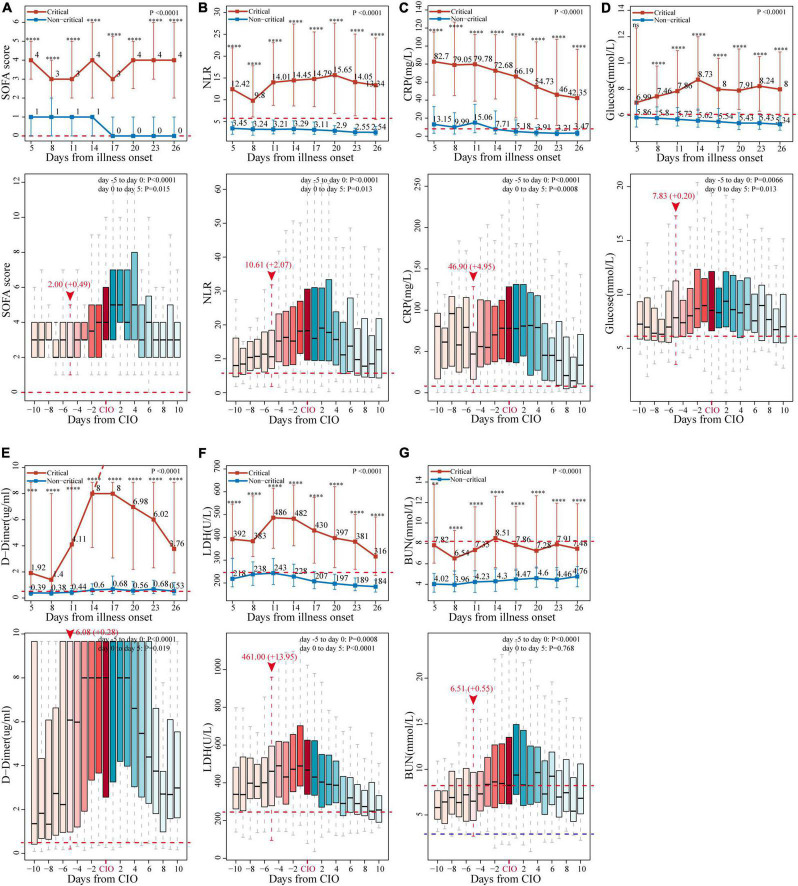
Change patterns of seven representative indicators in critical and non-critical COVID-19 patients. The dynamic changes of **(A)** SOFA score, **(B)** neutrophil-to-lymphocyte ratio (NLR), **(C)** C-reactive protein (CRP), **(D)** glucose, **(E)** D-dimer, **(F)** lactate dehydrogenase (LDH), and **(G)** blood urea nitrogen (BUN), starting from illness onset between critical and non-critical patients (line chart), and those starting from critical illness onset (CIO) in critical patients (boxplot). The horizontal red dotted line and the horizontal blue dotted line represent the upper and lower limits of the reference value range of each indicator, respectively. In line chart, the results are reported as median (IQR), *p*-values of the comparison of each marker at each timepoint and the overall change trend between critical and non-critical patients have also been displayed (**p < 0.01, ***p < 0.001, ****p < 0.0001, and ns for no significance). The values of D-dimer after day 14 exceeded the upper limit of detection, as indicated by the dashed line. In the boxplot, the day of “burning point” is designated as day 0 and highlighted by vertical red dotted line and red arrow, above which are indicator’s median value at the day of “burning point” and its average daily increment from “burning point” to CIO, they are expressed in the form of median value (+ increment per day), *p*-values for the change of the seven markers in critical patients from days 5 to 0 and days 0 to 5 have also been given, respectively. All the indicators’ values and *p*-values were calculated and analyzed by the linear mixed model, which have been adjusted for age and sex.

We compared the change patterns of SOFA score and 46 laboratory variables in 296 critical and 854 non-critical patients from illness onset to 26 days later by plotting line charts (Figure 3 and Supplementary Figures S5–7). Most of the indicators were substantially higher in critical patients than in non-critical patients during the whole observation period, including a sustained high level of the SOFA score, inflammatory biomarkers [NLR, CRP, white blood cells (WBCs), NEUs, procalcitonin (PCT), and ferritin], coagulation indices [D-dimer, prothrombin time (PT), international normalised ratio (INR), and activated partial thromboplastin time (APTT)], organ dysfunction indicators [LDH; creatine kinase (CK), brain natriuretic peptide (BNP), creatine kinase muscle-brain isoform (CK-MB), myoglobin, hypersensitive cardiac troponin I (hsTNI); total bilirubin (TBIL), direct bilirubin (DBIL), alkaline phosphatase (ALP), aspartate aminotransferase (AST), Globin, total bile acid (TBA), γ-glutamyl transpeptidase (GGT), Alanine aminotransferase (ALT); blood urea nitrogen (BUN), cystatin C (Cys-C)]), and metabolism parameter (glucose). However, some indicators were persistently lower in critical patients than in their non-critical counterparts, and these indicators were indicative of immune damage (lymphocytes and eosinophils), coagulation disorder (platelets), impaired liver function (A/G), and malnutrition (hemoglobin, RBCs, TP, prealbumin, and albumin). Importantly, several laboratory markers started to rise or drop on the 8th (7th–9th) day after illness onset in critical patients, such as NEUs, NLR, D-dimer, LDH, BUN, PCT, myoglobin, globin (all rose), lymphocyte, albumin, A/G, and HDL-C (all dropped) (Figure 3, Supplementary Figures S5–7, and Supplementary Notes).

### Second Alert: A “Burning Point”—Identified by Studying the Dynamic Changes Before Critical Illness Onset in Patients With Critical Illness

We further examined the dynamic changes of these 47 indicators before and after the CIO in 296 critical patients. As shown in Figure 3 and Supplementary Figures S5–7, boxplots showed the dynamic changes in laboratory findings and SOFA score starting from the CIO in critical patients. Indicators, including SOFA score, NLR, CRP, PCT, ferritin (four inflammatory biomarkers), lymphocytes (immune indicator), D-dimer (coagulation index), LDH (organ dysfunction variable), glucose (metabolic indicator), TP, and albumin (two nutrient indicators), were abnormal from the beginning and started to progress substantially and continuously on the 5th day before the CIO. Some other indices, including WBCs, NEUs, hemoglobin, RBCs, platelet, BUN, CK, BNP, and DBIL, were virtually within the normal range from the beginning but become abnormal upon approaching CIO, which indicated the same change in the pattern within the 5 days before CIO. Moreover, indicators, including PT, INR, and ALP, were not only constantly within the reference value range but also began to change persistently on the 5th day before CIO (all in Figure 3 and Supplementary Figures S5–7). Based on the above facts, the “burning point” was identified to be on the 5th day before CIO, a critical turning point indicating that CIO was only 5 days away, at which several indicators would experience further clear and continuous changes. This “burning point” appeared 12 (IQR, 7–17) days after illness onset (Figure 1B). As shown in Table 2 and Supplementary Table S8, LMM analysis revealed 26 out of 47 indicators changed significantly and continuously within 5 days before CIO, involving aspects of hematology, coagulation function, inflammation, energy and metabolism, cardiac, liver, and renal functions. The seven most significant and representative indicators were selected as reference indicators for clinical judgment. They were SOFA score [value, 2; increase per day (I/d), +0.49; *p* < 0.001], NLR (value, 10.61; I/d, +2.07; *p* < 0.0001), CRP (value, 46.9; I/d, +4.95 mg/L; *p* < 0.0001), glucose (value, 7.83; I/d, +0.2 mmol/L; *p* = 0.0066), D-dimer (value, 6.08; I/d, +0.28 μg/L; *p* < 0.0001), LDH (value, 461; I/d, +13.95 U/L; *p* = 0.0008), and BUN (value, 6.51; I/d, +0.55 mmol/L; *p* < 0.0001), each being presented as median value at the 5th day before CIO plus average daily increment between burning point and CIO [in square bracket] (Figure 3 and Table 2). The dynamic changes of all these 47 indicators after the CIO have been shown in Supplementary Table S9.

**TABLE 2:** Dynamic changes of SOFA score and laboratory findings before the critical illness onset (CIO).

Variables	Day-5	Day-3	Day-1	Day-0 Critical illness onset	Estimate	Std. error	Pr (> |t|)
**Representative variables, median (IQR)**							
SOFA score	2.00 (2.00–4.00)	3.00 (3.00–4.00)	4.00 (2.00–5.00)	4.00 (3.00–6.00)	0.492	0.033	<0.0001
NLR	10.61 (7.25–17.99)	16.33 (7.96–24.03)	18.19 (11.58–27.00)	18.29 (9.59–30.55)	2.068	0.264	<0.0001
CRP, mg/L	46.90 (16.23–73.52)	54.93 (35.62–111.10)	78.20 (41.03–111.97)	78.00 (37.36–128.24)	4.951	0.958	<0.0001
Glucose, mmol/L	7.83 (6.10–11.09)	8.01 (6.37–10.55)	8.96 (7.45–11.36)	8.50 (6.62–12.12)	0.201	0.074	0.0066
D-dimer, μg/mL	6.08 (1.01–8.50)	8.00 (1.93–8.50)	8.00 (3.73–8.50)	8.00 (2.60–8.50)	0.282	0.067	<0.0001
LDH, U/L	461.00 (278.50–594.50)	431.00 (287.00–616.00)	489.00 (383.00–702.50)	467.50 (339.00–625.50)	13.951	4.157	0.0008
BUN, mmol/L	6.51 (4.39–9.67)	8.36 (5.96–11.32)	8.45 (6.27–12.75)	8.25 (6.20–13.52)	0.547	0.096	<0.0001

*The linear mixed model has been adjusted for age and sex.*

*SOFA, sequential organ failure assessment; NLR, neutrophil-to-lymphocyte ratio; BUN, blood urea nitrogen; LDH, lactate dehydrogenase; CRP, C-reactive protein.*

## Discussion

In this study, on the basis of the analysis of 1,150 consecutive patients with COVID-19 who were admitted to Wuhan West Union Hospital from 12 January to 25 February 2020, we established a reliable baseline predictive model and developed an online tool to predict, at admission, the risk for the development to critical illness, which can be used as the first warning sign (the first alert). Moreover, in critical patients, we retrospectively identified a “burning point,” a warning sign that CIO was only 5 days away, and several indicators would experience significant and continuous changes. The “burning point” can serve as a second warning sign (the second alert), which can give clinicians precious time to take proactive measures before CIO. The two-checkpoint system can tell us “who” and “when” the critical illness will be developed in patients with COVID-19.

The predictors incorporated into the baseline predictive model were selected based on the random forest and LASSO regression analysis, which can provide a double guarantee for the selected predictors, ensuring the accuracy of the baseline model. Meanwhile, the model was translated into a nomogram system. Actually, the differentiating power of this nomogram scoring system was comparable to that of the aforementioned two models, yielding an AUC of 0.960 (95%CI, 0.941–0.972) (vs. 1.00 [95%CI, 1.00–1.00] vs. 0.971 [95%CI, 0.955–0.981]) in the training set and an AUC of 0.958 (95%CI, 0.936–0.980) (vs. 0.963 [95%CI, 0.941–0.986] vs. 0.956 [95%CI, 0.934–0.978]) in the testing set. The accuracy of this model was also fully validated by the internal 10,000 bootstrap and external testing set through the H–L test and calibration plots. The sensitivity analysis in the training (*p* = 0.43) and testing set (*p* = 0.26) further proved the good performance of this nomogram model. Furthermore, the DCA and clinical impact curves verified that this model worked effectively in supporting clinical decision-making. The nomogram system contained seven risk factors, including age, SOFA score, NLR, D-dimer, LDH, INR, and pneumonia area. All of them are easily obtained since they are included in the essential examinations at admission. Several studies (8, 23–25) have demonstrated that advanced age was an independent risk factor for death in patients with COVID-19. A higher SOFA score at admission was associated with increased odds of in-hospital death for patients with COVID-19 (15). Previous studies (11, 15, 26–28) showed that NLR, D-dimer, LDH, BUN, troponin, CRP, and PCT were risk predictors for the fatal outcome related to COVID-19. INR was reportedly higher in deceased patients than in convalescent patients with COVID-19 (29). Overall, the risk factor-based nomogram model is simple, effective, and amenable to clinical application, especially when transformed into a web-risk calculator, which can serve as the first alert for predicting critical illness in patients with COVID-19.

In addition, the longitudinal data analysis of critical and non-critical patients with COVID-19 demonstrated that almost all indicators showed conspicuous differences between those two groups, and several laboratory markers started to rise or drop on the 8th (7th–9th) day after illness onset in critical patients, supporting the hypothesis that the acute phase starts from the 7th to 10th day after illness onset of COVID-19, as proposed by a previous study (30). Collectively, differences in the aforementioned laboratory markers between critical and non-critical populations suggested that critical patients experienced long term coagulopathy, inflammatory activation, lymphocyte exhaustion, malnutrition, metabolic disorders, myocardial injury, liver dysfunction, and kidney injury. These findings can help us gain insight into the pathogenesis of COVID-19 and distinguish between critical and non-critical patients.

Moreover, we further looked into the dynamic changes in these 47 indicators before and after the CIO in 296 critical patients. The median time from illness onset to CIO was 17.0 (IQR, 12.0–22.0) days. We found that, prior to CIO, critical patients also suffered from severe coagulopathy (elevated D-dimer and declined PLT), inflammatory activation (elevated NEUs), lymphocyte exhaustion, myocardial damage (ascendant LDH and BNP), impaired liver function (elevated TBIL, AST, GGT, and ALT), kidney injury (ascendant BUN and Cys-C), malnutrition (reduced TP, albumin, and hemoglobin), and metabolic disorders (elevated glucose). Most importantly, we noticed that many laboratory markers started to have further and continuous changes on the 5th day before CIO. It indicates a turning point, at which the patient’s condition began to deteriorate before the CIO appeared. We designated this point as the “burning point,” which occurred 12 (IQR, 7–17) days after illness onset. This “burning point” corresponded exactly to a point in the early acute phase of COVID-19 proposed by Lin et al. (30). Furthermore, results of LMM revealed that 26 out of 47 indicators changed significantly and continuously within the 5 days before CIO, covering almost all the aspects of the above-mentioned abnormities. For clinical application, we selected the seven most significant and representative indicators as reference indicators and calculated their median values at the “burning point” (at the 5th day before CIO) and their average daily increments from “burning point” to CIO. These indicators were SOFA score, LDH, BUN (two organ-dysfunction indicators), CRP (inflammatory biomarkers), NLR (immune indicator), glucose (metabolism index), and D-dimer (coagulation indicator). In practice, we can judge whether a patient has passed the “burning point” on the basis of the time after illness onset, the value of each indicator at the “burning point” and its daily change increment. The appearance of a “burning point” indicates that CIO is only 5 days away, which can serve as the second alert before critical illness developed in patients with COVID-19.

Although the vaccine against COVID-19 is in full swing (31–33), there are still no special and effective treatments (34, 35). Intensifying multidisciplinary treatments, such as enhanced nutritional support, anticoagulation [low-molecular weight heparin (LMWH)], anti-inflammatory (γ-globulin, etc.), respiratory support (mechanical ventilation), and replacement therapy [continuous renal replacement therapy (CRRT)], are adopted to save lives of critical patients with COVID-19 (2, 36, 37). But the implementation of the above-mentioned intensive treatments usually started after the occurrence of critical illness. A recent study (30) about COVID-19 proposed that early initiation of intravenous γ-globulin and LMWH anticoagulant therapy was effective in improving the prognosis of patients with COVID-19. Since the “burning point” in this study represented the starting point at which the patient’s condition began to deteriorate before CIO, the high-risk period between the “burning point” and CIO might provide a precious time window for earlier intensive care and multidisciplinary interventions, thereby avoiding the aggravation to critical illness and improving survival.

Our study had several limitations. First, it was a single-center study. However, consecutively enrollment, large sample size (1,224), and low exclusion rate (74/1,224) reduce bias to some extent. Second, emerging SARS-CoV-2 variants characterized by increased transmissibility and decreased pathogenicity changed the landscape of the pandemic (38). However, considering that the mechanism of critical illness caused by different SARS-CoV-2 variant strains is similar to severe inflammatory syndrome (39), our predictive model may be used to predict the risk of critical illness due to infection by these variants and even other similar diseases. In addition, the methods section in our study has a greater reference value for similar studies and can be generalized to other critical diseases. Third, since all data were from China, the conclusion should be further validated in other countries.

## Conclusion

In conclusion, the baseline risk factors-based nomogram (the first alert) can be employed at admission to identify the high-risk patients who might progress to critical illness. During hospitalization, the “burning point” (the second alert) could be identified in patients with COVID-19 based on the time after illness onset, the value of each indicator at the “burning point,” and their daily change increments. The appearance of the “burning point” indicates that CIO was only 5 days away. The two sequential alerts allow early identification of deterioration of patients’ condition, which is critical in optimizing medical intervention and reducing the mortality rate of patients with COVID-19.

## Data Availability Statement

The original data presented in this study are included in the article/Supplementary Material, further inquiries can be directed to the corresponding author/s.

## Ethics Statement

The studies involving human participants were reviewed and approved by Institutional Review Board of Medical Ethics Committee of Union Hospital, Huazhong University of Science and Technology. Written informed consent for participation was not required for this study in accordance with the national legislation and the institutional requirements.

## Author Contributions

YJ and ZY designed the study. JX, HL, FW, GM, and LD collected and summarized the clinical data. ZY, XT, and CD checked all the data. XH, KW, ZY, and ZW cleaned and analyzed all data. ZY, MZ, XT, and KW drafted the manuscript. YJ, JX, and XH revised the final manuscript. YJ was the guarantor and attested that all listed authors meet authorship criteria and no others meeting the criteria have been omitted. All authors approved the final draft of the manuscript.

## Conflict of Interest

The authors declare that the research was conducted in the absence of any commercial or financial relationships that could be construed as a potential conflict of interest.

## Publisher’s Note

All claims expressed in this article are solely those of the authors and do not necessarily represent those of their affiliated organizations, or those of the publisher, the editors and the reviewers. Any product that may be evaluated in this article, or claim that may be made by its manufacturer, is not guaranteed or endorsed by the publisher.

## Footnotes

1
https://covid19.who.int
