# Supplementary material for: Predictive Risk Factors at Admission and a “Burning Point” During Hospitalization Serve as Sequential Alerts for Critical Illness in Patients With COVID-19
Source: Front Med (Lausanne). 2022 Jul 4;9:816314. doi: 10.3389/fmed.2022.816314 (PMC9291637; doi:10.3389/fmed.2022.816314)
Supplement: Supplementary file 1 [file Data_Sheet_1.docx]

**Supplementary materials**

**Predictive risk factors at admission and a "burning point" during hospitalization serve as sequential alerts for critical illness in COVID-19 patients**

Zhengrong Yin MD^a†^, Mei Zhou MD^a†^, Juanjuan Xu PhD^a†^, Kai Wang MD^b†^, Xingjie Hao PhD^b†^, Xueyun Tan MD^a^, Hui Li MS^a^, Fen Wang MS^a^, Chengguqiu Dai MS^b^, Guanzhou Ma PhD^a^, Zhihui Wang MS^c^, Limin Duan MD^a^, Yang Jin PhD^a^*

- **Abbreviations**
- **Supplementary methods**

Details of data processing and model construction

Model validation and comparison

- **Supplementary notes**

Definition of various organ injuries

Dynamic changes of 47 indicators in critical and non-critical COVID-19 patients

- **Table S1.** Outlier rate and missing rate for each variable and the missing rate of variables in each participant
- **Table S2.** The proportion of variance for the first 10 principal components in PCA analysis
- **Table S3.** Variables selected in Random Forest prediction model
- **Table S4.** Variables selected in LASSO prediction model
- **Table S5.** Comparison of the AUC values (C-statistics) among different models
- **Table S6.** The observed and predicted events of critical illness by nomogram model for each quantile in the testing set
- **Table S7.** Baseline laboratory results of critical and non-critical patients with COVID-19
- **Table S8.** Dynamic changes of other laboratory findings before the critical illness onset (CIO)
- **Table S9.** Dynamic changes of SOFA score and laboratory findings after the critical illness onset (CIO)
- **Figure S1.** Principle component analysis
- **Figure S2.** LASSO Regression analysis
- **Figure S3.** Hosmer-Lemeshow test of three prediction models in the testing set
- **Figure S4.** Online prediction tool developed based on the nomogram to predict, at admission, the risk of developmet to critical illness in COVID-19 patients
- **Figure S5.** Change pattern of hemotologic and coagulation indicators
- **Figure S6.** Change pattern of inflammatory, energy&metabolism and Cardiac function indicators
- **Figure S7.** Change pattern of Liver and Renal function indicators

**Abbreviations**

Q2±3IQR, median ± 3*interquartile range; AUC, area under the receiver operating characteristic curve; COPD, chronic obstructive pulmonary disease; ARDS, acute respiratory distress syndrome; CT, computed tomography; SOFA, Sequential Organ Failure Assessment; WBC, white blood cell; RBC, red blood cell; PLT, platelet; NLR, neutrophil to lymphocyte ratio; TBIL, total bilirubin; DBIL, direct bilirubin; ALT, Alanine aminotransferase; AST, Aspartate aminotransferase; ALP, Alkaline phosphatase; GGT, γ-glutamyl transpeptidase; TP, total protein; A/G, Albumin/globin; TBA, total bile acid; Tch, total cholesterol; TG, triglyceride; HDL-C, high density lipoprotein cholesterol; LDL-C, low density lipoprotein cholesterol; BUN, blood urea nitrogen; UA, uric acid; Cys-C, cystatin C; CK, creatine kinase; LDH, lactate dehydrogenase; CRP, C-reactive protein; PCT, procalcitonin; PT, prothrombin time; INR, international normalised ratio; APTT, activated partial thromboplastin time; FIB, fibrinogen; TT, thrombin time; BNP, brain natriuretic peptide; CK-MB, creatine kinase muscle-brain isoform; hsTNΙ, hypersensitive cardiac troponin Ι

**Supplementary methods**

**Details of data processing and model construction**

In our study cohort, 1150 adult COVID-19 patients and 87 variables for each participant were collected to construct baseline prediction models. Three physicians and four trained medical students checked the collected data for accuracy. For continuous variables, any observation that lied outside the range of median ± 3 * interquartile ranges (IQR) was considered to be an outlier and recorded as a missing value. The outlier rate and missing rate for each variable and participants were shown in **Table S1,** respectively. To ensure the data integrity and avoid potential selection bias, variables or participants with missing rate of less than 40% were all included, thus six variables (i.e. PCT, Ferritin, BNP, myoglobin, CK-MB, hsTNI) and 32 patients were excluded. Subsequently, 81 variables including 2 demographic variables, 12 comorbidity variables, 14 symptom variables, 42 laboratory finding variables, 10 imaging features variables, and the variable of time from illness onset to admission were analyzed and 1118 patients remained (**Table S1**). We used the random forest machine learning method to impute the remaining missing values. This method use the proximity matrix computed with the completed observations to impute the missing values [1]. For continuous variables, the imputed value was the weighted average of the completed observations, where the weights were the proximities. For categorical variables, the imputed value was the category with the largest average proximity. Given the imputed dataset, we conducted principal component analysis (PCA) to explore the distribution of participants and evaluated the important variables related to the critical illness with the R package “factoextra” [2]. The singular value decomposition method was utilized to examine the covariance matrix computed by normalized variables and the first 10 principal components (PCs) were extracted (**Table S2**). The first four PCs were plotted for participants to explore the distribution of sample and were plotted for variables to access the potential variables related to critical illness outcome. Participants were labelled as outlier and excluded subsequently if their first two PCs were out the corresponding range of median ± 3*IQR and no cases exceeded this range and were excluded in this process (**Figure S1 A-D**). PCA showed that the first principal component (PC1) was mainly composed of laboratory indicators, including CRP, SOFA score, NLR, LDH etc. (**Figure S1 E**), rather than comorbidities, and performed better in distinguishing the condition (critical or non-critical) of participants (**Figure S1 B**).

To construct the prediction model for critical illness outcome, we randomly divided the remaining participants (N=1118) into a training set (70% of participants, N=783 [non-critical/critical: 587/196]) and a testing set (30%, N=335 [non-critical/critical: 241/94]). Firstly, machine learning based Random forest model and Least Absolute Shrinkage and Selection Operator (LASSO) logistic regression were applied to develop the prediction models on training dataset.

We trained the random forest model with all the 81 variables by using the R package “randomForest” and then extracted the importance of each variable, measured by the Mean Decrease Gini index[3]. We then chose the top 20 important features (variables) to construct the final random forest prediction model (**Table S3**). LASSO regression penalizes the absolute size of the coefficients of a regression model by the λ parameter (ranging from 0 to 1) to improve the prediction accuracy and interpretability. With larger penalties (λ), the coefficients of weaker predictors will be shrunk to zero, so that only the strongest prediction variables remained in the model. We conducted a 10-fold cross-validation to train model and optimal λ was selected based on the area under the receiver operating characteristic curve (*AUC*) with the R package “glmnet” [4] (**Figure S2**). We then constructed the final LASSO prediction model with the variables (n=19) having the strongest prediction power determined by the optimal λ (**Table S4**)**.**

Finally, we chose the variables, appearing in both the random forest prediction model and LASSO prediction model, as candidate predictors to construct multivariable logistic prediction model. We performed univariable logistic analysis for each of these candidate predictors, and computed the corresponding odds ratio (*OR*) with 95% confidence interval (*CI*) and *AUC*. To avoid the co-linearity problem, we chose one of the two predictors with the maximum *AUC*, if the spearmen rank correlation coefficient between predictors was ≥0.60. We then performed multivariable logistic regression analysis with the remaining predictors. To enhance the clinical application of the logistic prediction model, we established a nomogram with the results from the multivariable logistic regression by using the package “rms”[5], which was then used to construct a web-based risk calculator

(<https://hust-covid19.shinyapps.io/Critical-illness-Predictive-Tool/>) (**Figure S4**).

**Model validation and comparison**

The distinguishing power of these three models was assessed by the C-statistics (also AUC [area under curve]) on both training and testing datasets. For internal validation, we estimated the C-statistics with 95% confidence interval (CI) by using 10,000 bootstrap resamples to reduce overfitting bias. The difference in C-statistics between these models was analyzed by non-parametric bootstrap method with 10,000 resamples (**Table S5**). The Hosmer-Lemeshow (H-L) test (**Table S6, Figure S3**) and calibration plot comparing the predictive and actual probabilities of critical illness were utilized to validate the predictive performance of these models. Besides, we performed decision curve analysis (DCA), which quantifies the net benefits and cost benefit ratio, to evaluate the clinical benefits of these models with the R package “Decision Curve” [6]. Sensitivity analysis was finally conducted to assess the influence of the incomplete observations appearing in the predictors in nomogram model by leaving the incomplete cases out.

**Supplementary notes**

**Definition of various organ injuries**

The diagnostic criteria for acute kidney injury were based on the KDIGO clinical practice guidelines [7]. Cardiac injury was confirmed if the serum level of hypersensitive cardiac troponin I (hsTNI) was above the upper limit of the reference range (>28 pg/mL) [8]. Liver injury was diagnosed according to the elevated bilirubin and aminotransferase [9].

**Dynamic changes of 47 indicators in critical and non-critical COVID-19 patients**

As shown in **Figure 3, Figure S5-7**, we discovered that critical patients, not non-critical patients, had a consistently high level of SOFA score, indicating that severe sepsis persisted in critical patients [10, 11]. Compared with non-critical patients, critical patients had a persistently higher level of abnormal inflammatory indices, such as neutrophils, CRP, and PCT, suggesting that critical patients were in a lasting state of inflammation activation. At the early stage of the disease, lymphopenia and decreased eosinophils could be found in both two groups but the lymphocyte level was much lower in critical patients. Afterwards, they gradually returned to be normal in non-critical patients, but remained abnormal in critical patients. These findings implied that lymphocytes were continuously depleted and immune response was impaired in critical patients, this was consistent with a previous study which showed that restored lymphocytes, eosinophils could serve as the predictors for the recovery of COVID-19 patients [12]. As for the coagulation index, D-dimer of critical patients was dramatically elevated and the level of PLT was decreased while this was not the case in non-critical patients, indicating the presence of severe and long-term coagulation disorder in the critical patients. Moreover, we found that critical patients had cardiac dysfunction (higher LDH, hsTNI, BNP, and myoglobin), hepatic dysfunction (elevated DBIL, TBIL, AST, GGT, ALT and decreased TP, albumin, prealbumin, A/G) and renal dysfunction (increased BUN, Cys-C) during the whole hospital-stay period. However, non-critical patients only had mild liver dysfunction. A recent study [13] suggested that persistent inflammation and coagulation disorders might be closely related to myocardial damage. The RBC and hemoglobin in most critically ill patients were initially within the normal range, but then gradually dropped to abnormal level. In contrast, the majority of non-critical patients had RBC and hemoglobin within the normal range throughout the hospital stay. Decreased levels of TP, albumin, prealbumin, HDL-C, RBC and hemoglobin suggested excessive body consumption and malnutrition in critical patients. Therefore, nutritional support for critical patients should be strengthened. Besides, only 31.1% of critical patients had diabetes, suggesting that lasting high fasting blood glucose levels in critical patients might be a result of stress responses to acute SARS-CoV-2 infection. It was reported that both acute and chronic hyperglycemia could aggravate COVID-19 patients’ condition by up-regulating the expression of ACE2 [14]. **Tables**

| **Table S1. Outlier rate and missing rate for each variable and the missing rate of variables in each participant** | | | | |
| --- | --- | --- | --- | --- |
| **Missing variables** | | **Outlier, n/N (%) Q2±3*IQR** | | **Number of cases, n (%) (N=1150)** |
| **Demographics** | | | | |
| Age | | - | | 0 (0) |
| Sex | | - | | 0 (0) |
| **Clinical characteristics** | | | | |
| Time from illness onset to admission | | - | | 0 (0) |
| **Initial symptoms** | | | | |
| Fever | | - | | 30 (2.6) |
| Highest temperature | | - | | 84 (7.3) |
| Sore throat | | - | | 59 (5.1) |
| Fatigue | | - | | 46 (4.0) |
| Myalgia | | - | | 54 (4.7) |
| Cough | | - | | 37 (3.2) |
| Sputum production | | - | | 46 (4.0) |
| Chest tightness | | - | | 46 (4.0) |
| Dyspnea | | - | | 51 (4.4) |
| Running nose | | - | | 55 (4.8) |
| Vomiting | | - | | 50 (4.3) |
| Nausea | | - | | 50 (4.3) |
| Diarrhea | | - | | 47 (4.1) |
| Headache | | - | | 52 (4.5) |
| Asymptomatic | | - | | 30 (2.6) |
| **Comorbidities** | | | | |
| Hypertension | | - | | 17 (1.5) |
| Diabetes | | - | | 17 (1.5) |
| Coronary heart disease | | - | | 17 (1.5) |
| Cerebrovascular disease | | - | | 17 (1.5) |
| Malignancy | | - | | 17 (1.5) |
| Chronic bronchitis | | - | | 17 (1.5) |
| Asthma | | - | | 17 (1.5) |
| COPD | | - | | 17 (1.5) |
| Kidney disease | | - | | 17 (1.5) |
| Liver disease | | - | | 17 (1.5) |
| Others | | - | | 17 (1.5) |
| Number of comorbidities | | - | | 17 (1.5) |
| **Complications** | | | | |
| Sepsis | | - | | 1 (0.1) |
| ARDS | | - | | 0 (0) |
| Acute liver injury | | - | | 1 (0.1) |
| Acute cardiac injury | | - | | 1 (0.1) |
| Acute kidney injury | | - | | 1 (0.1) |
| **Baseline CT features** | | | | |
| Pneumonia area (lesion ratio to lung) | | - | | 41 (3.6) |
| Uni-/Bilateral pneumonia | | - | | 41 (3.6) |
| Central/Peripheral (lesion location) | | - | | 41 (3.6) |
| Consolidation | | - | | 41 (3.6) |
| Patchy exudation | | - | | 41 (3.6) |
| Ground-glass opacity | | - | | 41 (3.6) |
| White lung | | - | | 41 (3.6) |
| Pleural effusion | | - | | 41 (3.6) |
| Lymph node enlargement | | - | | 41 (3.6) |
| **SOFA score at admission** | | 14/1137 (1.2) | | 30 (2.6) |
| **Baseline Laboratory findings** | | | | |
| WBC | | 23/1116 (2.1) | | 57 (5) |
| RBC | | 2/1114 (0.2) | | 38 (3.3) |
| Hemoglobin | | 5/1114 (0.4) | | 41 (3.6) |
| PLT | | 3/1114 (0.3) | | 39 (3.4) |
| Neutrophil count | | 28/1115 (2.5) | | 63 (5.5) |
| Lymphocyte count | | 3/1116 (0.3) | | 37 (3.2) |
| NLR | | 64/1115 (5.7) | | 99 (8.6) |
| Monocyte count | | 12/1114 (1.1) | | 48 (4.2) |
| Eosinophil count | | 27/1114 (2.4) | | 63 (5.5) |
| Basophil count | | 22/1114 (2) | | 58 (5) |
| TBIL | | 30/1103 (2.7) | | 77 (6.7) |
| DBIL | | 40/1103 (3.6) | | 87 (7.6) |
| ALT | | 41/1104 (3.7) | | 87 (7.6) |
| AST | | 40/1106 (3.6) | | 84 (7.3) |
| ALP | | 29/1065 (2.7) | | 114 (9.9) |
| GGT | | 85/1094 (7.8) | | 141 (12.3) |
| TP | | 5/1104 (0.5) | | 51 (4.4) |
| Albumin | | 0/1104 (0) | | 46 (4) |
| Globin | | 8/1104 (0.7) | | 54 (4.7) |
| Albumin/globin | | 1/1104 (0.1) | | 47 (4.1) |
| Prealbumin | | 0/1051 (0) | | 99 (8.6) |
| TBA | | 55/1056 (5.2) | | 149 (13) |
| Tch | | 3/719 (0.4) | | 434 (37.7) |
| TG | | 22/719 (3.1) | | 453 (39.4) |
| HDL-C | | 4/719 (0.6) | | 435 (37.8) |
| LDL-C | | 1/719 (0.1) | | 432 (37.6) |
| Creatinine | | 48/1101 (4.4) | | 97 (8.4) |
| BUN | | 53/1098 (4.8) | | 105 (9.1) |
| UA | | 20/1101 (1.8) | | 69 (6) |
| Cys-C | | 55/1006 (5.5) | | 199 (17.3) |
| Glucose | | 69/1083 (6.4) | | 136 (11.8) |
| CK | | 64/925 (6.9) | | 289 (25.1) |
| LDH | | 28/1098 (2.6) | | 80 (7) |
| CO_2_ | | 1/1006 (0.1) | | 145 (12.6) |
| CRP | | 0/1077 (0) | | 73 (6.3) |
| PCT | | 62/734 (8.4) | | 478 (41.6) |
| Ferritin | | 0/195 (0) | | 955 (83) |
| D-dimer | | 127/882 (14.4) | | 395 (34.3) |
| PT | | 21/1007 (2.1) | | 164 (14.3) |
| INR | | 21/1008 (2.1) | | 163 (14.2) |
| APTT | | 9/1008 (0.9) | | 151 (13.1) |
| FIB | | 0/1006 (0) | | 144 (12.5) |
| TT | | 28/1008 (2.8) | | 170 (14.8) |
| BNP | | 20/301 (6.6) | | 869 (75.6) |
| Myoglobin | | 37/349 (10.6) | | 838 (72.9) |
| CK-MB | | 36/352 (10.2) | | 834 (72.5) |
| hsTNΙ | | 41/386 (10.6) | | 805 (70) |
| **Missing rate of variables in each participant** | | | | |
| 0% | - | | 262 (22.8) | |
| 0-1% | - | | 0 (0) | |
| 1-5% | - | | 280 (24.3) | |
| 5-10% | - | | 345 (30) | |
| 10-20% | - | | 143 (12.4) | |
| 20-30% | - | | 52 (4.5) | |
| 30-40% | - | | 36 (3.1) | |
| >40% | - | | 32 (2.8) | |
| **Note:** The result of Missing value includes the sample data judged as outlier plus the missing value existing in original data | | | | |

| **Table S2. The** **proportion of variance explained by each of the first 10 principal components in PCA analysis** | | | | | | | | | | |
| --- | --- | --- | --- | --- | --- | --- | --- | --- | --- | --- |
| **Item** | **Principal Components (****PC)** | | | | | | | | | |
|  | PC1 | PC2 | PC3 | PC4 | PC5 | PC6 | PC7 | PC8 | PC9 | PC10 |
| Proportion of Variance (%) | 10.8 | 5.3 | 4.3 | 3.7 | 3.3 | 2.9 | 2.6 | 2.5 | 2.3 | 2.1 |
| Cumulative Proportion (%) | 10.8 | 16.2 | 20.4 | 24.1 | 27.4 | 30.3 | 32.8 | 35.3 | 37.6 | 39.7 |

| **Table S3. Variables selected in Random Forest prediction model** | |
| --- | --- |
| **Variables** | **Mean Decrease of Gini index** |
| SOFA score | 65.413 |
| NLR | 27.131 |
| D-dimer | 26.216 |
| LDH | 24.480 |
| CRP | 17.974 |
| Neutrophil count | 13.641 |
| Lymphocyte count | 12.093 |
| PLT | 9.777 |
| WBC | 9.375 |
| Prealbumin | 9.260 |
| BUN | 8.857 |
| Age | 8.793 |
| CK | 8.689 |
| HDL-C | 7.984 |
| Glucose | 7.849 |
| INR | 7.674 |
| Pneumonia area (lesion ratio to lung) | 7.327 |
| Tch | 7.317 |
| LDL-C | 7.012 |
| DBIL | 6.590 |

| **Table S4. Variables selected in LASSO prediction model** | |
| --- | --- |
| **Variable** | **Beta** |
| Age | 0.008 |
| Coronary heart disease | 0.396 |
| Cerebrovascular disease | 1.559 |
| Other underlying diseases | 0.199 |
| SOFA score | 1.236 |
| Neutrophil count | 0.103 |
| NLR (neutrophil to lymphocyte ratio) | 0.022 |
| ALP (Alkaline phosphatase) | 0.001 |
| Creatinine | -0.011 |
| LDH (lactate dehydrogenase) | 0.002 |
| CRP (C-reactive protein) | 0.010 |
| D-dimer | 0.305 |
| INR (international normalised ratio) | 1.553 |
| Highest temperature | -0.089 |
| Dyspnea | 0.108 |
| Pneumonia area (lesion ratio to lung): small *vs.* medium | 0.820 |
| Pneumonia area (lesion ratio to lung): small *vs.* large | 1.216 |
| Ground glass | 0.136 |
| White lung | 1.182 |

| **Table S5. Comparison of the AUC values (c-statistics) among different models** | | | | |
| --- | --- | --- | --- | --- |
|  | **Random forest & LASSO** | **Random forest & Nomogram** | **LASSO & Nomogram** | **Nomogram & sensitivity analysis** |
| **Training set,**  **P value** | <.001 | <.001 | <.001 | .43 |
| **Testing set,**  **P value** | .63 | .73 | .55 | .26 |
| **Note:** 10,000 non-parametric bootstrap test was used for the comparison of AUC values | | | | |

| **Table S6. The observed and predicted events of critical illness for each quantile in testing set** | | | | | | | | | | | |
| --- | --- | --- | --- | --- | --- | --- | --- | --- | --- | --- | --- |
|  | **Quantile** | | | | | | | | | | ***P* value** |
|  | **0-10th** | **10-20th** | **20-30th** | **30-40th** | **40-50th** | **50-60th** | **60-70th** | **70-80th** | **80-90th** | **90-100th** |  |
| **Random forest prediction model** | | | | | | | | | | | |
| Sample size | 35 | 32 | 35 | 32 | 34 | 33 | 33 | 34 | 33 | 34 | .445 |
| Observed events | 0 | 0 | 1 | 1 | 2 | 1 | 5 | 17 | 31 | 33 |  |
| Predicted events | 0.063 | 0.224 | 0.745 | 1.349 | 3.045 | 5.345 | 9.047 | 17.545 | 24.771 | 30.960 |  |
| **LASSO prediction model** | | | | | | | | | | | |
| Sample size | 34 | 33 | 34 | 33 | 34 | 33 | 33 | 34 | 33 | 34 | .744 |
| Observed events | 0 | 0 | 0 | 2 | 0 | 3 | 6 | 18 | 30 | 32 |  |
| Predicted events | 0.187 | 0.380 | 0.632 | 1.110 | 2.061 | 3.921 | 7.997 | 16.649 | 27.238 | 33.240 |  |
| **Nomogram** | | | | | | | | | | | |
| Sample size | 34 | 33 | 34 | 33 | 34 | 33 | 33 | 34 | 33 | 34 | .863 |
| Observed events | 0 | 0 | 1 | 0 | 1 | 2 | 8 | 20 | 26 | 33 |  |
| Predicted events | 0.059 | 0.147 | 0.309 | 0.698 | 1.581 | 3.604 | 9.099 | 19.449 | 29.400 | 33.657 |  |
| **Note:** *P* value was calculated by Hosmer-Lemeshow test. | | | | | | | | | | | |

**Table S7. Baseline laboratory results of critical and non-critical patients with COVID-19**

| **Variables** | **All patients, [n=1150]** | **Non-critical patients,**  **[n=854]** | **Critical patients,**  **[n=296]** | ***P* value** |
| --- | --- | --- | --- | --- |
| **Baseline Laboratory findings, median (IQR) or mean (±SD)** | | | | |
| White blood cells, x10^9^/L | 5.75 (4.34, 7.55) | 5.33 (4.22, 6.88) | 7.39 (5.12, 10.26) | < 0.0001 |
| Red blood cells , x10^12^/L | 4.10 (±0.58) | 4.10 (±0.55) | 4.09 (±0.67) | 0.950 ^*^ |
| Hemoglobin, g/L | 125.65 (±17.36) | 125.22 (±16.00) | 126.90 (±20.73) | 0.212 ^*^ |
| Platelet, x10^9^/L | 211.0 (153.00, 278.00) | 222.00 (166.00, 287.75) | 166.00 (115.00, 239.00) | < 0.0001 |
| Neutrophil count, x10^9^/L | 3.96 (2.82, 5.97) | 3.68 (2.65, 5.10) | 6.29 (3.96, 8.87) | <0.0001 |
| Lymphocyte count, x10^9^/L | 0.99 (0.68, 1.36) | 1.09 (0.80, 1.47) | 0.65 (0.46, 0.91) | <0.0001 |
| Monocyte count, x10^9^/L | 0.37 (0.27, 0.51) | 0.39 (0.29, 0.53) | 0.30 (0.20, 0.48) | <0.0001 |
| Eosinophil count, x10^9^/L | 0.02 (0, 0.07) | 0.03 (0.01, 0.08) | 0.01 (0, 0.02) | <0.0001 |
| Basophil count, x10^9^/L | 0.01 (0.01, 0.02) | 0.01 (0.01, 0.02) | 0.01 (0, 0.03) | 0.247 |
| Total bilirubin, μmol/L | 10.80 (8.20, 14.30) | 10.30 (7.80, 13.30) | 12.40 (9.05, 17.50) | <0.0001 |
| Direct bilirubin, μmol/L | 3.30 (2.50, 4.70) | 3.20 (2.30, 4.30) | 4.40 (3.10, 5.90) | <0.0001 |
| Alanine aminotransferase, U/L | 31.00 (19.00, 49.00) | 29.00 (19.00, 48.00) | 33.40 (23.00, 52.00) | 0.0025 |
| Aspartate aminotransferase, U/L | 30.00 (22.00, 43.00) | 28.00 (20.00, 40.00) | 40.00 (27.25, 55.00) | <0.0001 |
| Alkaline phosphatase, U/L | 57.00 (44.00, 73.00) | 55.00 (44.00, 69.00) | 65.50 (48.00, 87.25) | <0.0001 |
| γ-glutamyl transpeptidase, U/L | 28.00 (18.00, 45.00) | 26.00 (17.00, 43.00) | 36.00 (24.00, 59.00) | <0.0001 |
| Total protein, g/L | 63.10 (±6.06) | 63.47 (±5.86) | 62.04 (±6.51) | 0.0006 ^*^ |
| Albumin, g/L | 31.48 (±5.53) | 32.42 (±5.47) | 28.83 (±4.81) | <0.0001 ^*^ |
| Globin, g/L | 31.54 (±5.22) | 30.99 (±4.92) | 33.10 (±5.72) | <0.0001 ^*^ |
| Albumin/globin | 1.00 (0.80, 1.20) | 1.10 (0.90, 1.30) | 0.90 (0.70, 1.10) | <0.0001 |
| Prealbumin, mg/L | 139.90 (92.55, 208.05) | 156.30 (111.40, 225.90) | 90.50 (66.15, 132.17) | <0.0001 |
| Total bile acid, μmol/L | 2.70 (1.70, 4.40) | 2.60 (1.60, 4.30) | 3.20 (2.00, 5.10) | 0.0004 |
| Total cholesterol, mmol/L | 3.96 (3.40, 4.57) | 4.02 (3.44, 4.58) | 3.83 (3.27, 4.47) | 0.028 |
| Triglyceride, mmol/L | 1.29 (1.00, 1.69) | 1.27 (0.99, 1.67) | 1.33 (1.10, 1.72) | 0.054 |
| High density lipoprotein cholesterol, mmol/L | 0.90 (0.76, 1.09) | 0.92 (0.78, 1.11) | 0.84 (0.71, 1.01) | <0.0001 |
| low density lipoprotein cholesterol, mmol/L | 2.36 (±0.77) | 2.39 (±0.74) | 2.26 (±0.85) | 0.037 ^*^ |
| Creatinine, μmol/L | 69.52 (±18.99) | 68.24 (±18.30) | 73.48 (±20.52) | 0.0001 ^*^ |
| Blood urea nitrogen, mmol/L | 4.47 (3.48, 6.01) | 4.25 (3.26, 5.45) | 5.97 (4.25, 8.10) | <0.0001 |
| Uric acid, μmol/L | 240.70 (183.90, 303.40) | 242.60 (189.10, 303.40) | 233.80 (173.40, 304.60) | 0.280 |
| Cystatin-C, mg/L | 0.84 (0.72, 0.97) | 0.81 (0.71, 0.93) | 0.91 (0.78, 1.07) | <0.0001 |
| Glucose, mmol/L | 6.00 (5.30, 7.29) | 5.84 (5.24, 6.86) | 6.90 (5.87, 8.48) | <0.0001 |
| Creatine kinase, U/L | 73.00 (46.00, 126.00) | 68.00 (44.00, 111.75) | 94.00 (55.00, 186.50) | <0.0001 |
| CO_2_, mmol/L | 25.13 (±5.01) | 25.55 (±4.90) | 23.90 (±5.15) | <0.0001 ^*^ |
| C-reactive protein, mg/L | 24.39 (4.36, 65.35) | 13.70 (3.13, 43.72) | 69.17 (34.15, 109.97) | <0.0001 |
| Procalcitonin, ng/mL | 0.08 (0.05, 0.14) | 0.06 (0.04, 0.12) | 0.16 (0.10, 0.29) | <0.0001 |
| Ferritin, ng/ml | 543.28 (228.13, 1128.53) | 400.24 (195.84, 805.12) | 1047.6 (569.77, 2084.3) | <0.0001 |
| Prothrombin time, s | 13.26 (±1.20) | 13.08 (±1.05) | 13.81 (±1.41) | <0.0001^*^ |
| Activated partial thromboplastin time, s | 36.00 (32.50, 40.10) | 35.50 (32.40, 39.20) | 37.30 (32.85, 42.40) | 0.001 |
| Fibrinogen, g/l | 4.16 (±1.25) | 4.15 (±1.14) | 4.18 (±1.51) | 0.813 ^*^ |
| Thrombin time, s | 15.62 (±1.17) | 15.53 (±1.06) | 15.90 (±1.42) | 0.0002^*^ |
| Brain natriuretic peptide, pg/ml | 30.90 (12.90, 94.60) | 21.90 (5.00, 66.15) | 59.60 (30.90, 130.10) | <0.0001 |
| Myoglobin, ng/ml | 45.30 (31.27, 76.90) | 39.30 (27.40, 62.20) | 75.20 (42.60, 132.50) | <0.0001 |
| Creatine kinase muscle-brain isoform, ng/ml | 0.80 (0.50, 1.30) | 0.70 (0.40, 1.10) | 1.00 (0.70, 1.80) | <0.0001 |
| hypersensitive cardiac troponin I, ng/L | 4.50 (1.90, 10.30) | 3.90 (1.90, 8.10) | 7.55 (2.59, 16.45) | 0.0008 |

Note: Data were presented as n/N (%), median (IQR) or mean (±SD). P values were calculated by Mann-Whitney U test, χ² test, or Fisher’s exact, if not specified; * t-test. Abbreviations: IQR: interquartile range; SD: standard deviation.

**Table S8. Dynamic changes of other laboratory findings before the critical illness onset (CIO).**

| Variables | Day -5 | Day -3 | Day -1 | Day 0  Critical illness onset | Estimate | Std. Error | Pr (>\|t\|) |
| --- | --- | --- | --- | --- | --- | --- | --- |
| [Hematology](javascript:;), median (IQR) | | | | | | | |
| WBC, ×10^9^/L | 8.14 (6.39-10.73) | 9.23 (6.58-11.57) | 12.80 (9.48-15.07) | 11.12 (7.71-16.48) | 0.799 | 0.097 | <0.0001 |
| Neutrophil count, ×10^9^/L | 7.10 (5.12-9.82) | 8.29 (5.68-10.58) | 10.84 (7.30-13.75) | 9.96 (6.69-15.20) | 0.766 | 0.093 | <0.0001 |
| Lymphocyte count, ×10^9^/L | 0.73 (0.46-0.96) | 0.56 (0.42-0.75) | 0.53 (0.34-0.76) | 0.55 (0.36-0.81) | -0.026 | 0.006 | <0.0001 |
| Hemoglobin, g/L | 124.50 (114.00-141.75) | 125.00 (109.00-140.00) | 122.00 (104.00-133.00) | 118.50 (103.00-133.25) | -2.106 | 0.303 | <0.0001 |
| RBC, ×10^12^/L | 4.14 (3.57-4.51) | 3.95 (3.59-4.51) | 3.90 (3.36-4.32) | 3.93 (3.33-4.39) | -0.065 | 0.009 | <0.0001 |
| Monocyte count, ×10^9^/L | 0.38 (0.21-0.51) | 0.30 (0.17-0.53) | 0.32 (0.20-0.54) | 0.37 (0.22-0.54) | 0.008 | 0.005 | 0.107 |
| Eosinophil count, ×10^9^/L | 0.01 (0-0.02) | 0.01 (0-0.03) | 0.01 (0-0.02) | 0.01 (0-0.03) | 0.0002 | 0.0009 | 0.798 |
| Coagulation function, median (IQR) | | | | | | | |
| PLT, ×10^9^/L | 188.50 (121.75-241.50) | 134.00 (96.00-208.00) | 136.00 (85.00-215.00) | 141.50 (85.00-221.00) | -4.987 | 1.104 | <0.0001 |
| PT, s | 13.40 (12.70-14.25) | 14.35 (13.67-16.35) | 14.60 (13.50-16.40) | 14.50 (13.40-15.60) | 0.141 | 0.045 | 0.0016 |
| INR | 1.05 (0.99-1.12) | 1.15 (1.07-1.29) | 1.17 (1.07-1.31) | 1.16 (1.05-1.25) | 0.009 | 0.004 | 0.035 |
| TT, s | 15.70 (14.75-16.75) | 16.30 (14.70-17.30) | 15.95 (14.67-17.52) | 15.60 (14.60-16.60) | -0.038 | 0.045 | 0.393 |
| FIB, g/l | 4.01 (2.85-5.01) | 3.42 (2.39-4.69) | 3.85 (2.46-4.84) | 3.95 (2.80-5.01) | 0.014 | 0.032 | 0.657 |
| APTT, s | 34.05 (30.12-38.75) | 36.90 (32.70-41.80) | 37.25 (30.98-43.35) | 37.05 (32.12-41.70) | 0.064 | 0.167 | 0.702 |
| Inflammation, median (IQR) | | | | | | | |
| PCT, ng/mL | 0.14 (0.10-0.36) | 0.21 (0.12-0.49) | 0.38 (0.18-0.69) | 0.31 (0.13-0.62) | 0.026 | 0.014 | 0.066 |
| Ferritin, ng/ml | 1259.0 (935.9-1991.4) | 1980.0 (1378.0-2000.5) | 1232.4 (707.4-2000.5) | 1086.0 (540.1-2000.5) | -36.202 | 33.047 | 0.277 |
| Energy and metabolism, median (IQR) | | | | | | | |
| LDL-C, mmol/L | 2.28 (1.69-2.66) | 2.07 (1.55-2.69) | 2.23 (1.82-2.84) | 2.03 (1.45-2.77) | -0.030 | 0.015 | 0.051 |
| Tch, mmol/L | 3.81 (3.44-4.62) | 3.61 (3.06-4.26) | 4.08 (3.22-4.75) | 3.56 (3.05-4.53) | -0.032 | 0.018 | 0.086 |
| HDL-C, mmol/L | 0.80 (0.71-0.91) | 0.76 (0.64-0.87) | 0.76 (0.66-0.85) | 0.77 (0.64-0.93) | -0.010 | 0.006 | 0.104 |
| TG, mmol/L | 1.52 (1.15-2.17) | 1.34 (1.19-2.13) | 1.66 (1.25-2.01) | 1.35 (1.1-1.89) | -0.010 | 0.017 | 0.558 |
| CO_2_, mmol/L | 27.60 (23.45-29.60) | 27.30 (22.95-29.45) | 26.05 (21.60-31.68) | 27.30 (23.10-30.10) | 0.115 | 0.113 | 0.308 |
| Cardiac function, median (IQR) | | | | | | | |
| CK, U/L | 70.00 (41.00-179.75) | 94.00 (62.00-236.00) | 139.50 (63.50-395.50) | 111.50 (57.00-281.00) | 16.331 | 4.355 | 0.0002 |
| BNP, pg/ml | 49.15 (29.83-95.30) | 134.10 (64.28-218.35) | 131.75 (54.50-276.12) | 95.00 (38.90-230.45) | 13.250 | 6.386 | 0.039 |
| CK-MB, ng/ml | 1.00 (0.65-1.70) | 1.55 (1.02-2.70) | 1.25 (0.72-2.63) | 1.70 (0.90-3.10) | 0.115 | 0.061 | 0.063 |
| Myoglobin, ng/ml | 92.50 (37.65-192.55) | 77.70 (56.26-139.12) | 81.40 (50.73-168.95) | 84.30 (55.88-175.22) | 8.214 | 6.090 | 0.179 |
| hsTNΙ, ng/L | 16.20 (7.80-24.70) | 10.10 (1.38-40.85) | 11.40 (4.79-50.95) | 16.65 (6.88-53.85) | 1.232 | 1.522 | 0.420 |
| Liver function, median (IQR) | | | | | | | |
| TBIL, μmol/L | 11.90 (9.50-21.80) | 16.00 (10.95-21.33) | 14.25 (9.97-22.96) | 15.20 (10.55-23.25) | 0.649 | 0.179 | 0.0003 |
| DBIL, μmol/L | 4.20 (2.90-6.60) | 5.70 (3.30-8.40) | 4.85 (3.03-10.57) | 5.50 (3.60-8.30) | 0.402 | 0.082 | <0.0001 |
| ALP, U/L | 69.00 (52.00-92.00) | 71.00 (57.00-96.00) | 80.00 (63.00-103.50) | 79.00 (59.00-101.00) | 1.807 | 0.484 | 0.0002 |
| TP, g/L | 60.95 (57.00-65.78) | 61.80 (56.00-66.18) | 60.30 (56.10-64.47) | 59.40 (55.75-65.68) | -0.298 | 0.131 | 0.023 |
| Albumin, g/L | 27.25 (26.18-31.32) | 25.90 (23.62-30.45) | 26.85 (23.52-29.32) | 27.70 (23.90-30.10) | -0.202 | 0.093 | 0.030 |
| Prealbumin, mg/L | 119.00 (78.35-167.02) | 96.00 (59.40-136.30) | 100.70 (64.30-134.00) | 98.05 (70.00-135.82) | -0.820 | 0.903 | 0.364 |
| AST, U/L | 44.00 (23.40-58.00) | 36.00 (28.00-52.00) | 38.00 (28.00-61.00) | 34.00 (25.00-52.00) | -1.070 | 0.491 | 0.030 |
| Globin, g/L | 33.10 (28.95-37.10) | 32.75 (29.50-38.75) | 33.80 (30.10-37.50) | 32.15 (29.00-37.45) | -0.065 | 0.100 | 0.519 |
| A/G | 0.80 (0.70-1.00) | 0.78 (0.60-0.90) | 0.80 (0.70-0.92) | 0.80 (0.70-1.00) | -0.005 | 0.004 | 0.215 |
| TBA, μmol/L | 3.00 (2.00-4.40) | 2.90 (1.60-5.65) | 3.50 (1.60-5.80) | 3.10 (1.80-5.00) | 0.053 | 0.065 | 0.410 |
| GGT, U/L | 40.00 (25.25-65.00) | 47.00 (26.00-74.00) | 46.55 (29.02-83.00) | 42.50 (28.00-64.00) | -0.332 | 0.426 | 0.436 |
| ALT, U/L | 37.00 (21.00-56.00) | 40.00 (21.80-59.50) | 34.00 (22.50-57.00) | 37.00 (24.00-55.50) | -0.185 | 0.496 | 0.709 |
| Renal function, median (IQR) | | | | | | | |
| Cys-C, mg/L | 0.97 (0.81-1.21) | 0.98 (0.84-1.28) | 1.03 (0.87-1.39) | 0.97 (0.80-1.33) | 0.027 | 0.008 | 0.0007 |
| Creatinine, μmol/L | 67.05 (56.70-81.17) | 73.50 (59.95-86.05) | 72.20 (58.20-98.55) | 68.00 (55.50-90.05) | 1.053 | 0.505 | 0.038 |
| UA, μmol/L | 220.85 (118.37-298.15) | 234.90 (158.45-316.20) | 227.35 (143.90-299.40) | 213.15 (146.90-311.1) | 2.483 | 2.118 | 0.242 |

Note: The linear mixed model has been adjusted for age and sex.

Abbreviations: PLT, platelet; LDH, lactate dehydrogenase; RBC, red blood cell; WBC, white blood cell; PT, prothrombin time; INR, international normalised ratio; TT, thrombin time; FIB, fibrinogen; APTT, activated partial thromboplastin time; PCT, procalcitonin; LDL-C, low density lipoprotein cholesterol; Tch, total cholesterol; HDL-C, high density lipoprotein cholesterol; TG, triglyceride; CK, creatine kinase; BNP, brain natriuretic peptide; CK-MB, creatine kinase muscle-brain isoform; hsTNΙ, hypersensitive cardiac troponin I; TBIL, total bilirubin; DBIL, direct bilirubin; ALP, Alkaline phosphatase; TP, total protein; AST, Aspartate aminotransferase; A/G, Albumin/globin; TBA, total bile acid; GGT, γ-glutamyl transpeptidase; ALT, Alanine aminotransferase; Cys-C, cystatin C; UA, uric acid.

**Table S9. Dynamic changes of SOFA score and laboratory findings after the critical illness onset (CIO).**

| **Variables** | **Day 0**  **Critical illness onset** | **Day 1** | **Day 3** | **Day 5** | **Estimate** | **Std. Error** | **Pr (>\|t\|)** |
| --- | --- | --- | --- | --- | --- | --- | --- |
| **Representative variables** | | | | | | | |
| SOFA score | 4.00 (3.00-6.00) | 5.00 (3.00-7.00) | 4.00 (3.00-7.00) | 3.00 (2.00-5.00) | 0.109 | 0.045 | 0.015 |
| NLR | 18.29 (9.59-30.55) | 16.02 (9.79-30.83) | 17.78 (9.53-33.26) | 11.16 (5.61-20.79) | -0.594 | 0.238 | 0.013 |
| CRP, mg/L | 78.00 (37.36-128.24) | 77.66 (36.30-131.28) | 81.45 (21.15-120.77) | 45.34 (15.06-83.65) | -3.417 | 1.011 | 0.0008 |
| Glucose, mmol/L | 8.50 (6.62-12.12) | 8.30 (6.89-10.59) | 8.58 (6.57-11.76) | 9.06 (6.68-11.53) | 0.037 | 0.077 | 0.628 |
| D-dimer, μg/mL | 8.00 (2.60-8.50) | 8.50 (3.40-8.50) | 8.00 (3.97-8.50) | 5.47 (2.57-8.50) | -0.102 | 0.043 | 0.019 |
| LDH, U/L | 467.50 (339.00-625.50) | 430.50 (336.00-623.00) | 392.00 (283.00-547.00) | 291.00 (226.00-434.00) | -19.029 | 3.383 | <0.0001 |
| BUN, mmol/L | 8.25 (6.20-13.52) | 9.37 (7.25-14.92) | 8.21 (6.17-12.56) | 7.30 (5.06-10.93) | 0.026 | 0.090 | 0.768 |
| **[Hematology](javascript:;)** | | | | | | | |
| WBC, ×10^9^/L | 11.12 (7.71-16.48) | 11.82 (8.64-16.23) | 10.86 (7.02-14.85) | 8.30 (6.39-10.95) | -0.439 | 0.090 | <0.0001 |
| Neutrophil count, ×10^9^/L | 9.96 (6.69-15.20) | 10.79 (7.38-14.84) | 9.59 (6.56-13.31) | 6.95 (5.82-9.30) | -0.404 | 0.085 | <0.0001 |
| Lymphocyte count, ×10^9^/L | 0.55 (0.36-0.81) | 0.56 (0.30-0.91) | 0.49 (0.35-0.82) | 0.52 (0.35-0.89) | 0.002 | 0.006 | 0.748 |
| Hemoglobin, g/L | 118.50 (103.00-133.25) | 113.00 (96.00-124.00) | 108.00 (97.25-118.75) | 107.50 (82.00-119.00) | -2.504 | 0.274 | <0.0001 |
| RBC, ×10^12^/L | 3.93 (3.33-4.39) | 3.63 (3.10-4.07) | 3.52 (3.01-3.86) | 3.49 (2.64-3.89) | -0.077 | 0.009 | <0.0001 |
| Monocyte count, ×10^9^/L | 0.37 (0.22-0.54) | 0.31 (0.20-0.52) | 0.36 (0.23-0.57) | 0.36 (0.21-0.51) | -0.008 | 0.005 | 0.101 |
| Eosinophil count, ×10^9^/L | 0.01 (0-0.03) | 0.01 (0-0.05) | 0.01 (0-0.05) | 0.01 (0-0.04) | 0.00004 | 0.0009 | 0.964 |
| **Coagulation function** | | | | | | | |
| PLT, ×10^9^/L | 141.50 (85.00-221.00) | 114.00 (66.00-218.00) | 128.50 (64.00-215.50) | 145.50 (74.50-212.75) | -3.392 | 1.058 | 0.001 |
| PT, s | 14.50 (13.40-15.60) | 14.90 (13.90-15.83) | 14.50 (13.60-15.45) | 14.30 (13.35-15.60) | -0.024 | 0.039 | 0.536 |
| INR | 1.16 (1.05-1.25) | 1.19 (1.10-1.29) | 1.15 (1.06-1.26) | 1.14 (1.04-1.26) | -0.001 | 0.004 | 0.754 |
| TT, s | 15.60 (14.60-16.60) | 15.20 (14.60-16.30) | 15.30 (14.28-16.83) | 15.35 (14.65-15.88) | 0.0009 | 0.043 | 0.984 |
| FIB, g/l | 3.95 (2.80-5.01) | 3.75 (2.49-4.79) | 3.78 (2.67-4.76) | 3.78 (2.71-5.03) | -0.021 | 0.028 | 0.458 |
| APTT, s | 37.05 (32.12-41.70) | 37.00 (31.80-43.50) | 36.80 (33.35-41.95) | 36.10 (30.72-42.58) | 0.204 | 0.153 | 0.183 |
| **Inflammation** | | | | | | | |
| PCT, ng/mL | 0.31 (0.13-0.62) | 0.55 (0.26-1.04) | 0.29 (0.10-0.73) | 0.18 (0.07-0.59) | -0.006 | 0.012 | 0.640 |
| Ferritin, ng/ml | 1086.0 (540.1-2000.5) | 1542.7 (356.0-2000.5) | 1317.5 (733.01-1738.2) | 1433.4 (1327.7-2000.5) | -40.222 | 40.072 | 0.320 |
| **Energy and metabolism** | | | | | | | |
| LDL-C, mmol/L | 2.03 (1.45-2.77) | 1.82 (1.15-2.62) | 1.91 (1.11-2.27) | 1.98 (1.56-2.56) | 0.005 | 0.017 | 0.770 |
| Tch, mmol/L | 3.56 (3.05-4.53) | 3.46 (2.80-4.20) | 3.29 (2.99-4.05) | 3.31 (2.88-4.36) | -0.024 | 0.021 | 0.254 |
| HDL-C, mmol/L | 0.77 (0.64-0.93) | 0.79 (0.65-0.89) | 0.84 (0.72-1.02) | 0.77 (0.57-0.96) | -0.006 | 0.006 | 0.333 |
| TG, mmol/L | 1.35 (1.1-1.89) | 1.56 (1.07-1.92) | 1.58 (1.06-2.08) | 1.25 (0.99-1.66) | 0.011 | 0.017 | 0.540 |
| CO_2_, mmol/L | 27.30 (23.10-30.10) | 28.20 (24.50-31.37) | 29.70 (26.50-32.10) | 28.70 (26.52-32.65) | 0.601 | 0.108 | < 0.0001 |
| **Cardiac function** | | | | | | | |
| CK, U/L | 111.50 (57.00-281.00) | 182.00 (65.00-359.75) | 59.50 (38.25-167.00) | 43.00 (29.00-67.00) | -7.063 | 3.609 | 0.052 |
| BNP, pg/ml | 95.00 (38.90-230.45) | 84.40 (49.90-197.60) | 157.75 (61.85-274.65) | 128.20 (54.70-262.90) | 7.562 | 5.624 | 0.180 |
| CK-MB, ng/ml | 1.70 (0.90-3.10) | 2.10 (1.20-3.90) | 1.20 (0.80-2.10) | 0.80 (0.70-1.40) | -0.231 | 0.061 | 0.0002 |
| Myoglobin, ng/ml | 84.30 (55.88-175.22) | 105.10 (61.80-319.80) | 121.90 (55.40-230.90) | 81.95 (46.15-136.47) | -1.023 | 5.567 | 0.854 |
| hsTNΙ, ng/L | 16.65 (6.88-53.85) | 26.10 (7.60-55.50) | 26.85 (7.77-104.47) | 21.30 (9.30-68.70) | -1.811 | 2.548 | 0.478 |
| **Liver function** | | | | | | | |
| TBIL, μmol/L | 15.20 (10.55-23.25) | 15.80 (10.85-22.40) | 13.00 (8.50-19.20) | 14.55 (8.48-21.17) | -0.054 | 0.176 | 0.759 |
| DBIL, μmol/L | 5.50 (3.60-8.30) | 6.95 (4.40-10.62) | 5.50 (3.60-9.10) | 5.30 (3.10-10.40) | 0.034 | 0.084 | 0.685 |
| ALP, U/L | 79.00 (59.00-101.00) | 74.50 (61.75-99.50) | 77.00 (64.50-96.75) | 75.00 (54.00-100.00) | -0.278 | 0.449 | 0.536 |
| TP, g/L | 59.40 (55.75-65.68) | 59.40 (54.10-63.20) | 60.90 (55.78-65.60) | 61.10 (56.57-65.25) | -0.037 | 0.126 | 0.766 |
| Albumin, g/L | 27.70 (23.90-30.10) | 27.00 (23.60-30.00) | 27.40 (24.60-31.40) | 30.30 (26.50-32.90) | 0.330 | 0.081 | < 0.0001 |
| Prealbumin, mg/L | 98.05 (70.00-135.82) | 97.60 (69.50-144.40) | 114.90 (82.55-149.45) | 138.55 (98.92-166.55) | 3.794 | 0.856 | < 0.0001 |
| AST, U/L | 34.00 (25.00-52.00) | 29.30 (23.00-54.00) | 30.00 (21.00-44.50) | 26.00 (20.00-43.00) | 0.102 | 0.467 | 0.827 |
| Globin, g/L | 32.15 (29.00-37.45) | 31.20 (27.40-34.30) | 33.25 (27.78-36.98) | 31.60 (25.83-35.12) | -0.390 | 0.091 | < 0.0001 |
| A/G | 0.80 (0.70-1.00) | 0.80 (0.70-1.00) | 0.80 (0.70-1.00) | 0.90 (0.80-1.20) | 0.022 | 0.004 | < 0.0001 |
| TBA, μmol/L | 3.10 (1.80-5.00) | 2.85 (1.50-5.57) | 3.30 (1.80-6.00) | 3.60 (2.30-5.60) | 0.235 | 0.061 | 0.0001 |
| GGT, U/L | 42.50 (28.00-64.00) | 43.00 (27.50-70.00) | 46.00 (31.00-61.50) | 45.00 (31.00-77.00) | -0.285 | 0.545 | 0.601 |
| ALT, U/L | 37.00 (24.00-55.50) | 40.00 (25.50-53.00) | 31.00 (22.50-54.00) | 30.00 (18.00-54.00) | -0.190 | 0.489 | 0.698 |
| **Renal function** | | | | | | | |
| Cys-C, mg/L | 0.97 (0.80-1.33) | 1.03 (0.83-1.49) | 0.94 (0.78-1.49) | 0.96 (0.83-1.48) | 0.014 | 0.008 | 0.069 |
| Creatinine, μmol/L | 68.00 (55.50-90.05) | 71.40 (56.70-85.62) | 58.85 (48.15-80.15) | 61.00 (51.50-73.20) | -0.022 | 0.520 | 0.966 |
| UA, μmol/L | 213.15 (146.90-311.10) | 195.80 (138.12-275.15) | 165.70 (89.30-238.70) | 140.60 (118.90-209.05) | -5.176 | 1.732 | 0.003 |

**Note**: The linear mixed model has been adjusted for age and sex.

**Abbreviations**: SOFA, Sequential Organ Failure Assessment; PLT, platelet; NLR, neutrophil to lymphocyte ratio; BUN, blood urea nitrogen; LDH, lactate dehydrogenase; RBC, red blood cell; WBC, white blood cell; PT, prothrombin time; INR, international normalised ratio; TT, thrombin time; FIB, fibrinogen; APTT, activated partial thromboplastin time; CRP, C-reactive protein; PCT, procalcitonin; LDL-C, low density lipoprotein cholesterol; Tch, total cholesterol; HDL-C, high density lipoprotein cholesterol; TG, triglyceride; CK, creatine kinase; BNP, brain natriuretic peptide; CK-MB, creatine kinase muscle-brain isoform; hsTNΙ, hypersensitive cardiac troponin I; TBIL, total bilirubin; DBIL, direct bilirubin; ALP, Alkaline phosphatase; TP, total protein; AST, Aspartate aminotransferase; A/G, Albumin/globin; TBA, total bile acid; GGT, γ-glutamyl transpeptidase; ALT, Alanine aminotransferase; Cys-C, cystatin C; UA, uric acid.

**Figures**

**Figure S1. Principle component analysis**


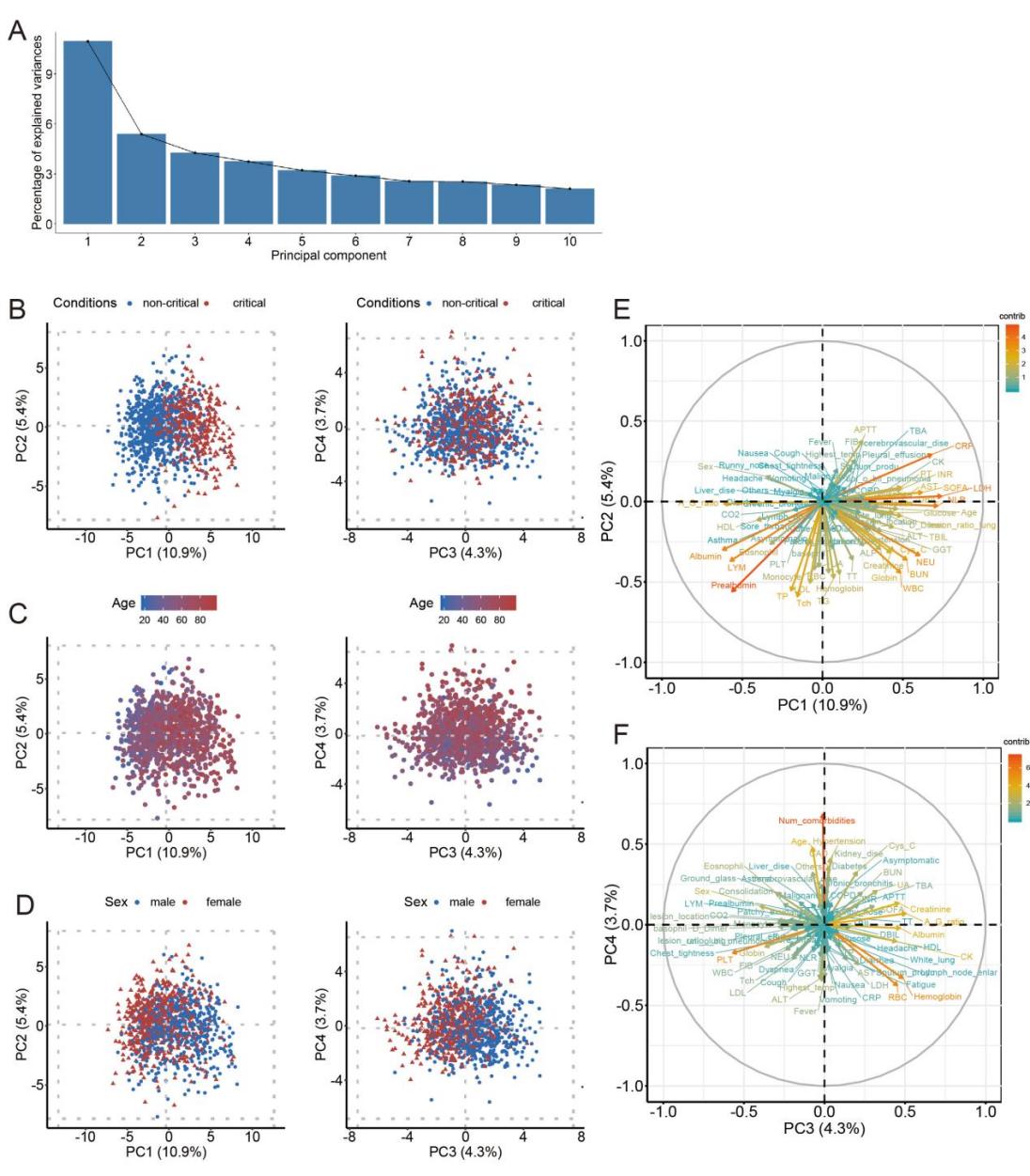


**(*A*)** Scree plot of principal component analysis (PCA). The percentage of variances explained by each of the selected 10 principal component were shown. PCA plots of the first four principle component (PC) for samples grouped by conditions **(*B*),** age **(*C*),** and sex **(*D*)** of participants; the grey dotted line represented median - 3*interquartile range, median, and median + 3*interquartile range respectively in both horizontal and vertical axis. PCA plots for Variables between PC1 and PC2 **(*E*)**, PC3 and PC4 **(*F*)**; positive correlated variables point to the same side of the plot, negative correlated variables was on the contrary; different colour for each variable indicated the contribution of a variable to a given principal component (in percentage).

**Figure S2. LASSO Regression analysis**


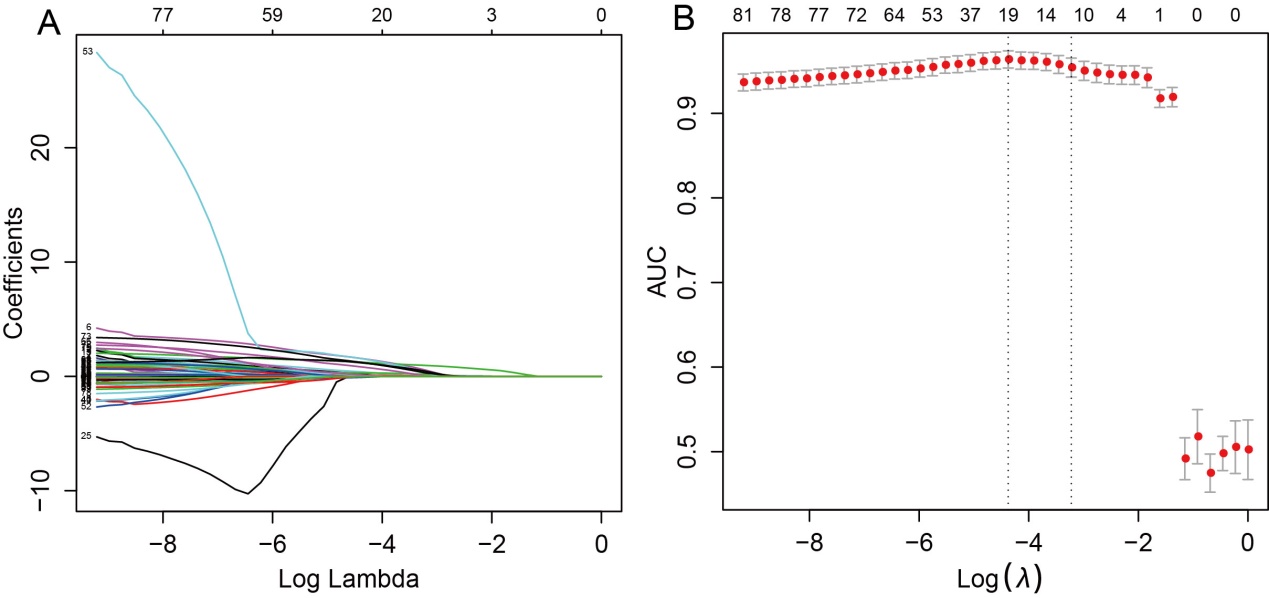


**(*A*)** Lasso coefficient profiles (y-axis) of the 81 variables. The lower x-axis indicated the Log Lambda (λ). The top x-axis indicated the average numbers of predictors. **(*B*)** Identification of the optimal λ in the Lasso regression with 10-fold cross-validation based on maximum area under the receiver operating characteristic (AUC). The vertical axis indicated AUC and horizontal axis indicated Log λ. Red dot with vertical bar represented average AUC and 95% CI for each model with a given λ.

**Figure S3. Hosmer-Lemeshow test of three prediction models in the testing set**


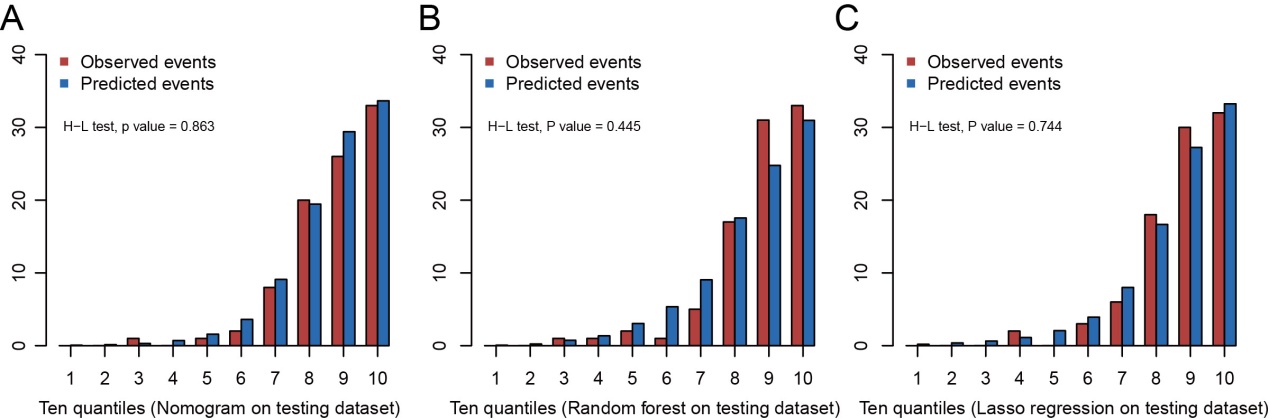


Bar plots of the observed events (red bar) and predicted events (blue bar) in each of the ten quantiles with the nomogram prediction model **(*A*)**, random forest prediction model **(*B*)** and Lasso regression prediction model **(*C*)** on testing set. H-L test refers to Hosmer-Lemeshow χ^2^ test.

**Figure S4: Online prediction tool developed based on the nomogram to predict, at admission, the risk of developmet to critical illness in COVID-19 patients**


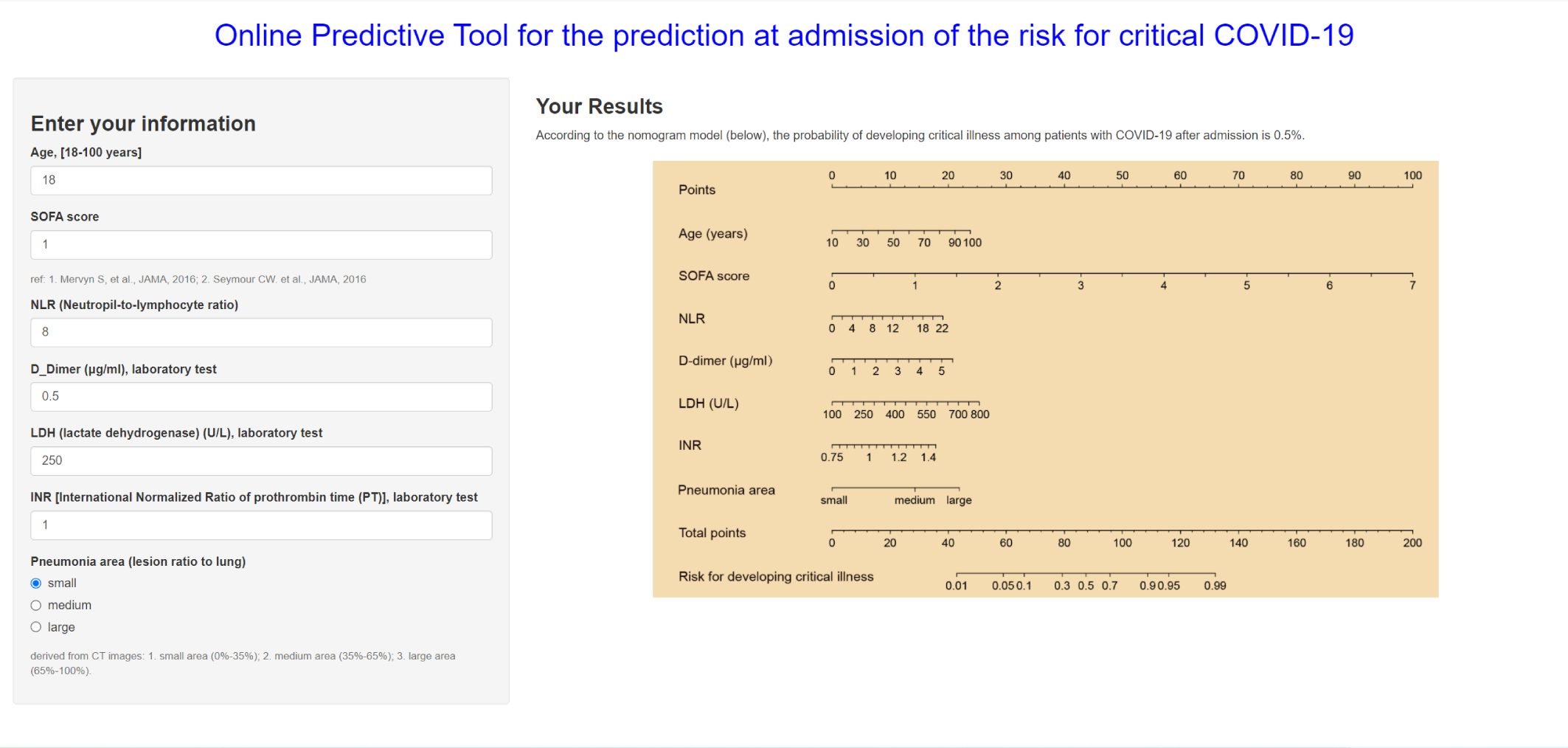


**Figure S5. Change pattern of hemotologic and coagulation indicators**


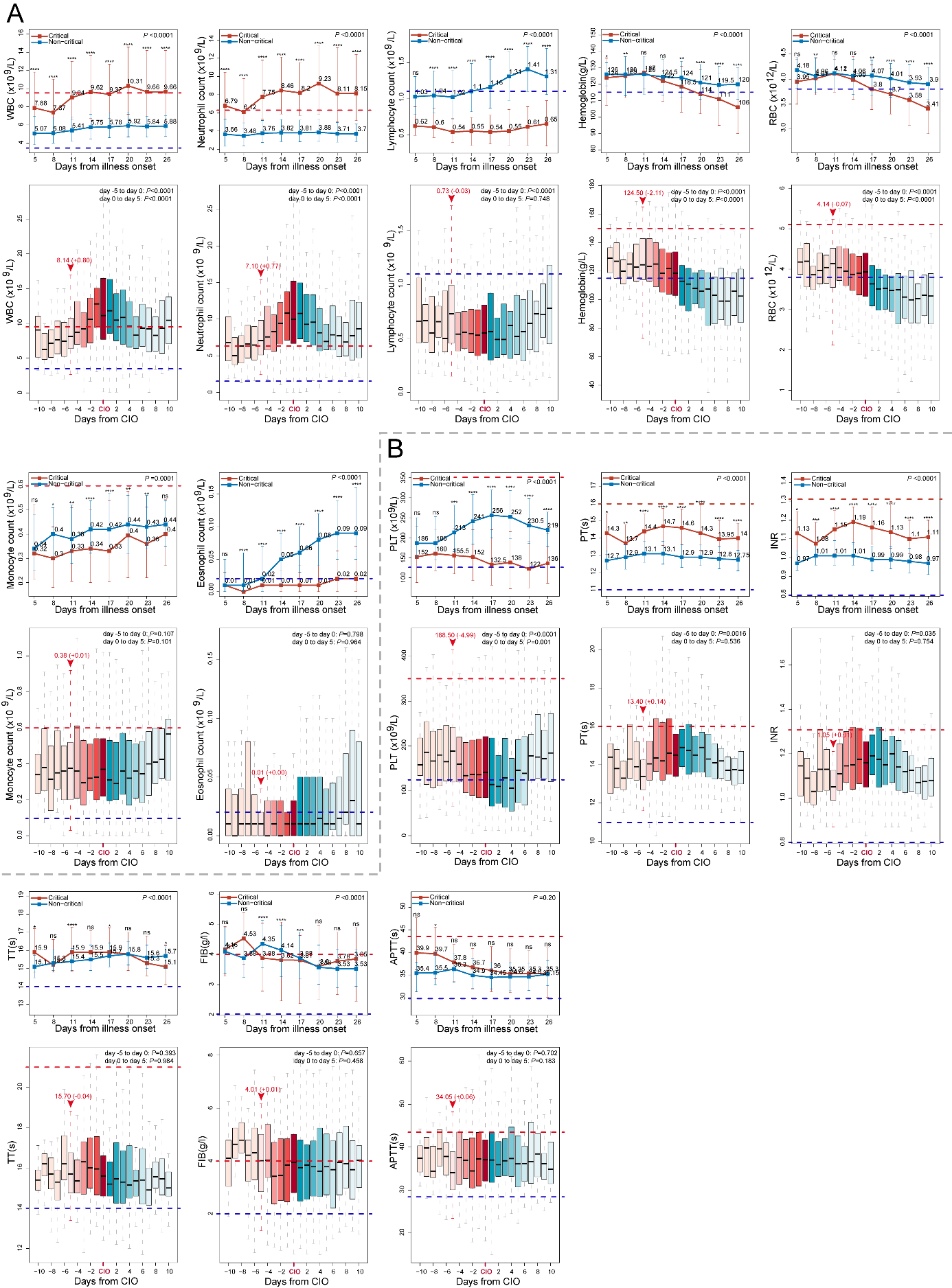


**(*A*)** The dynamic changes of hemotologic indicators starting from illness onset between critical and non-critical patients (line chart), and those starting from critical illness onset (CIO) in critical patients (boxplot). **(*B*)** The dynamic changes of coagulation indicators. The horizontal red dotted line and the horizontal blue dotted line represent the upper and lower limits of the reference value range of each indicator, respectively. In line chart, the results are reported as median (IQR). In the boxplot, the day of “burning point” is highlighted by vertical red dotted line and red arrow, above which are indicator's median value at the day of “burning point” and its average daily increment/decrement from “burning point” to CIO, they are expressed in the form of median (+increment) or median (-decrement).

**Figure S6. Change pattern of inflammatory, energy&metabolism and cardiac function indicators**


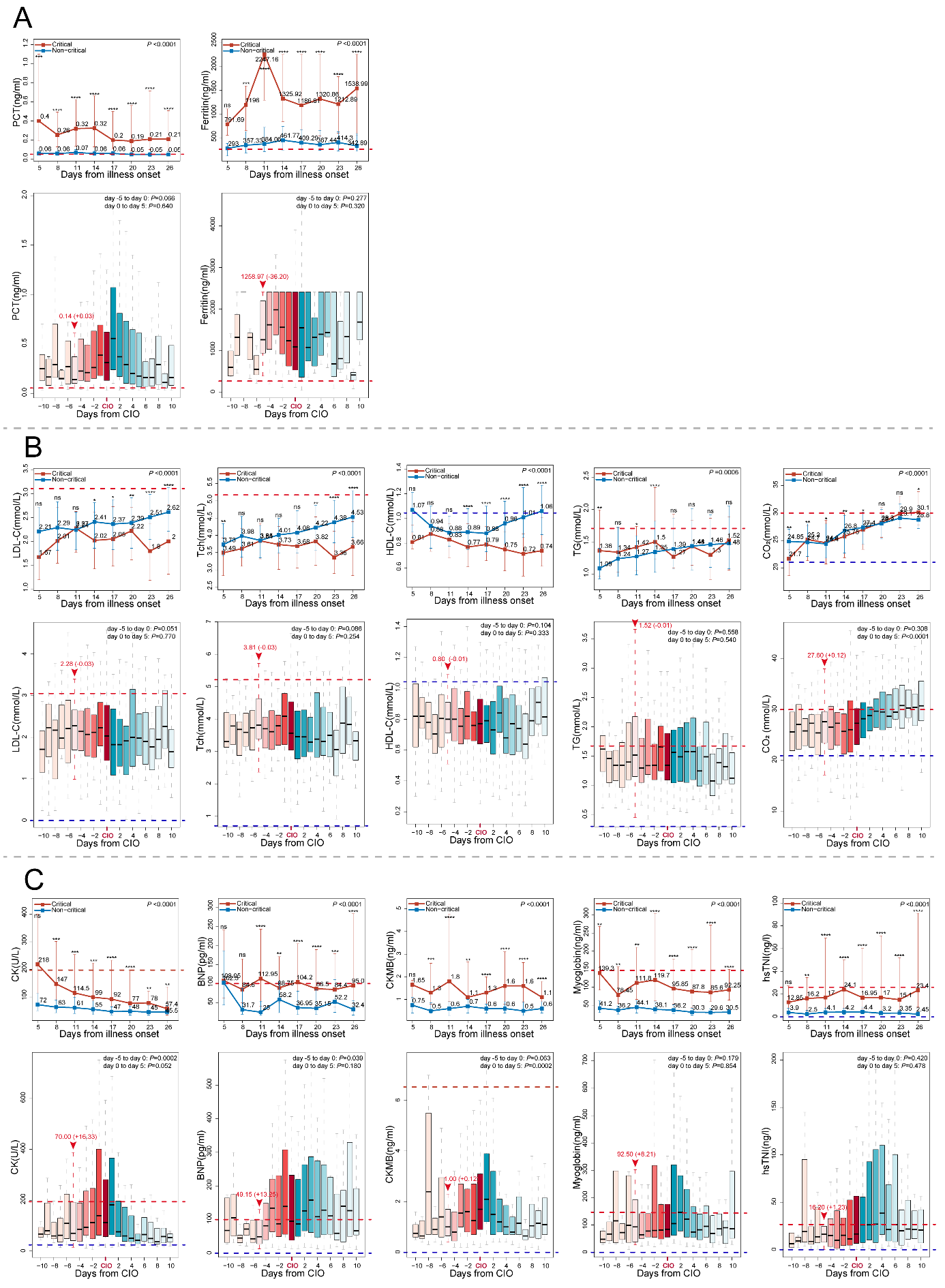


**(*A*)** The dynamic changes of inflammatory factors starting from illness onset between critical and non-critical patients (line chart), and those starting from CIO in critical patients (boxplot). The dynamic changes of energy&metabolism indicators **(*B*),** and cardiac function indicators **(*C*)**.

**Figure S7. Change pattern of liver and renal function indicators**


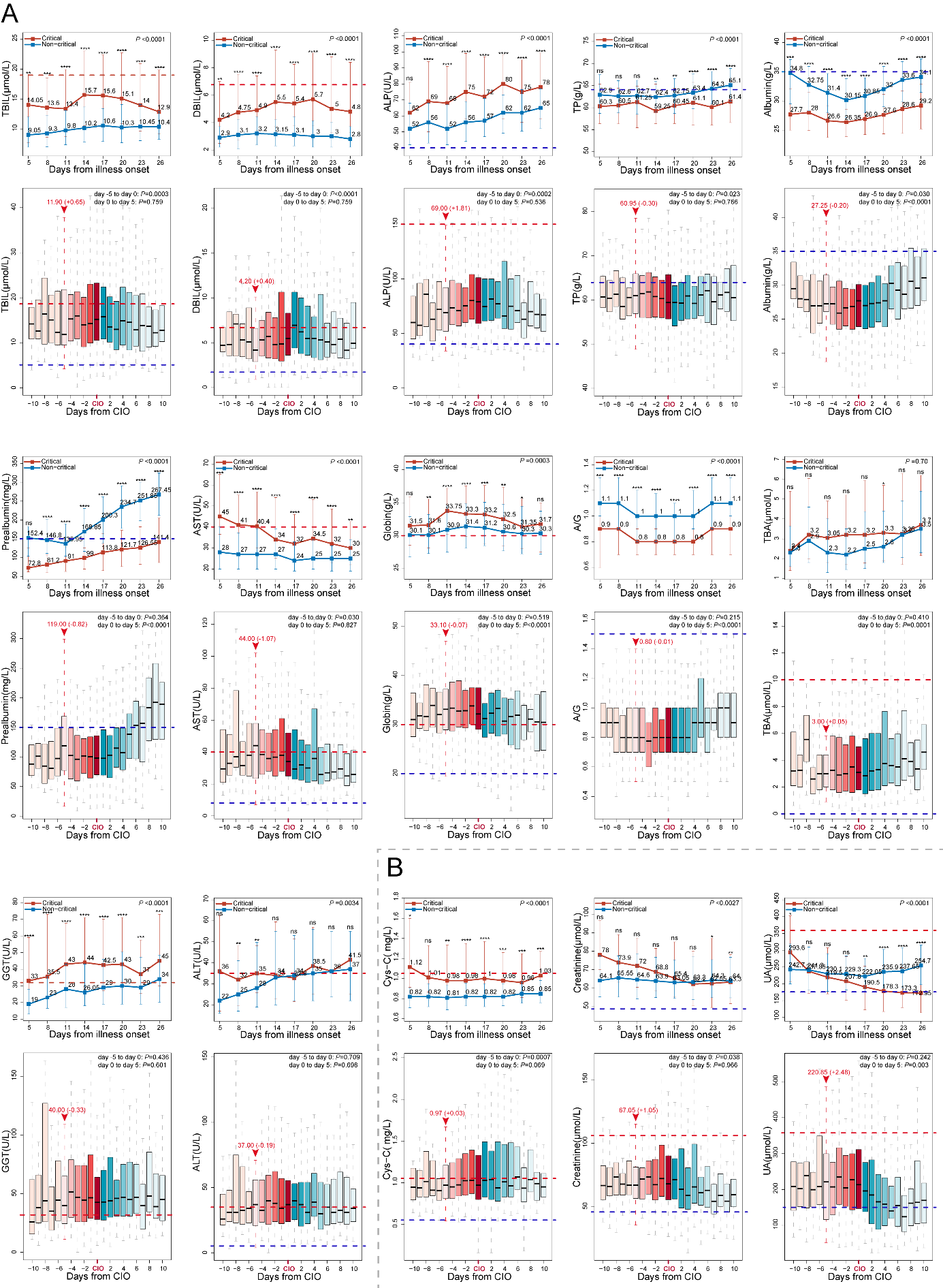


1. The dynamic changes of Liver function indicators starting from illness onset between critical and non-critical patients (line chart), and those starting from CIO in critical patients (boxplot). **(*B*)** The dynamic changes of renal function indicators.

**References**

1. L. B. Manual for Setting Up, Using, and Understanding Random Forest V4.0 [Available from: <https://www.stat.berkeley.edu/~breiman/Using_random_forests_v4.0.pdf>.

2. Kassambara A MF. Factoextra: extract and visualize the results of multivariate data analyses [1.0.7:[Available from: <https://CRAN.R-project.org/package=factoextra>.

3. Hong H XG, Hua Y. Variable selection using Mean Decrease Accuracy and Mean Decrease Gini based on Random Forest. *2016 7th IEEE International Conference on Software Engineering and Service Science (ICSESS)*. 2016.

4. Friedman J, Hastie T, Tibshirani R. Regularization Paths for Generalized Linear Models via Coordinate Descent. *Journal of statistical software*. 2010;33(1):1-22.

5. Harrell FE, Jr., Lee KL, Califf RM, Pryor DB, Rosati RA. Regression modelling strategies for improved prognostic prediction. *Stat Med*. 1984;3(2):143-52.

6. Kerr KF, Brown MD, Zhu K, Janes H. Assessing the Clinical Impact of Risk Prediction Models With Decision Curves: Guidance for Correct Interpretation and Appropriate Use. *J Clin Oncol*. 2016;34(21):2534-40.

7. Khwaja A. KDIGO clinical practice guidelines for acute kidney injury. *Nephron Clin Pract*. 2012;120(4):c179-84.

8. Yang X, Yu Y, Xu J, Shu H, Xia J, Liu H, et al. Clinical course and outcomes of critically ill patients with SARS-CoV-2 pneumonia in Wuhan, China: a single-centered, retrospective, observational study. *The Lancet Respiratory medicine*. 2020;8(5):475-81.

9. Chen T, Wu D, Chen H, Yan W, Yang D, Chen G, et al. Clinical characteristics of 113 deceased patients with coronavirus disease 2019: retrospective study. *BMJ*. 2020;368:m1091.

10. Zhou F, Yu T, Du R, Fan G, Liu Y, Liu Z, et al. Clinical course and risk factors for mortality of adult inpatients with COVID-19 in Wuhan, China: a retrospective cohort study. *Lancet*. 2020;395(10229):1054-62.

11. Lambden S, Laterre PF, Levy MM, Francois B. The SOFA score-development, utility and challenges of accurate assessment in clinical trials. *Crit Care*. 2019;23(1):374.

12. Chen R, Sang L, Jiang M, Yang Z, Jia N, Fu W, et al. Longitudinal hematologic and immunologic variations associated with the progression of COVID-19 patients in China. *J Allergy Clin Immunol*. 2020.

13. Shi S, Qin M, Cai Y, Liu T, Shen B, Yang F, et al. Characteristics and clinical significance of myocardial injury in patients with severe coronavirus disease 2019. *Eur Heart J*. 2020.

14. Bornstein SR, Rubino F, Khunti K, Mingrone G, Hopkins D, Birkenfeld AL, et al. Practical recommendations for the management of diabetes in patients with COVID-19. *The lancet Diabetes & endocrinology*. 2020.
